# Stakeholder Engagement in Competency Framework Development in Health Professions: A Systematic Review

**DOI:** 10.3389/fmed.2021.759848

**Published:** 2021-11-12

**Authors:** Breanna Lepre, Claire Palermo, Kylie J. Mansfield, Eleanor J. Beck

**Affiliations:** ^1^School of Medicine, Illawarra Health and Medical Research Institute, University of Wollongong, Wollongong, NSW, Australia; ^2^Monash Centre for Scholarship in Health Education, Monash University, Clayton, VIC, Australia

**Keywords:** competency frameworks, health professions, stakeholder engagement, education, competency development

## Abstract

Competency framework development in health professions has downstream implications for all relevant stakeholders, from the professionals themselves, to organisations, and most importantly end users of services. However, there is little guidance related to what stakeholders might be involved in the competency development process, and when. This review aimed to systematically review literature related to competency framework development methodology in health, to identify the breadth and purpose of key stakeholders commonly involved in the process. Studies were identified using five electronic databases (MEDLINE, PubMed, CINAHL, EMBASE, and ERIC) and a search of websites of organisations involved in curriculum or regulation using keywords related to competency frameworks. The total yield from all databases was 10,625 results, with 73 articles included in the final review. Most articles were from Australia (30%) and were conducted in the nursing (34%) profession. Unsurprisingly, practitioners (86%) and academics (75%) were typically engaged as stakeholders in competency framework development. While many competency frameworks were described as patient-focused, only 14 (19%) studies elected to include service users as stakeholders. Similarly, despite the multi-disciplinary focus described in some frameworks, only nine (12%) studies involved practitioners from other professions. Limiting the conceptualisation of competence to that determined by members of the profession itself may not provide the depth of insight required to capture the complexity of healthcare and address the needs of important stakeholder groups. Future methodology should attempt to engage a variety of relevant stakeholders such as external health professions and the community to match professional education to health service demands.

**Systematic Review Registration:** https://www.crd.york.ac.uk/PROSPERO/display_record.php?RecordID=128350

## Introduction

A competent workforce is a key element for effective health care systems (1). Competency based education recognises the need to match the health workforce to priorities for population health (2), and forms the basis of academic instruction and assessment for medicine, nursing and other health professions. A competency-based approach aims to maximise skill and resource utilisation and increase labour market efficiency (3). Competency frameworks provide an architectural blueprint for workforce development, fundamental in the delivery of person-centred care (4, 5). The frameworks are an increasingly important policy tool in defining knowledge, behaviour and skill in addition to professional standing for regulation and quality improvement purposes (4). They are often the basis for curricular development in health professional education programs and are also used as standards in accreditation of programs (6). Competency standards can be used in performance evaluation, professional development and for recruitment purposes, among other uses (2). Furthermore, there is significant positive correlation between frequency of use of competencies and perceived confidence in service provision by health professionals themselves (7).

There are many approaches to developing a competency framework (8). In health professions, the process of developing a competency framework is generally guided by the professional association (organisations representing the health profession members or regulatory body), with input from others, such as academics and practitioners (8). Specifically, members of medical, nursing and other health professions have traditionally defined their own professional competencies (8). There is limited guidance on competency framework development, including which stakeholder groups to involve in the process, when to include them and to what capacity. While competency framework development might be considered a specialised process, diversity in stakeholder engagement has been shown to increase the credibility of a competency framework for end users (8, 9). Health care practise is complex, and the development of competency frameworks in health professions requires strategies to capture and represent this reality and to meet patient and community needs. The outcomes and impacts of competency frameworks have implications for all stakeholder groups including students and education providers, practitioners, employers, policy makers, credentialing and certification organisations, and importantly, service users. For example the CanMEDS framework, which describes the abilities physicians require to meet the health care needs of the people they serve, has the overarching goal to improve patient care (6). Therefore, the perspectives of multiple stakeholders regarding the conceptualisation of competency and what should constitute the roles and functions of health professionals require consideration.

The general elements of competency development processes have been previously reviewed, and the critical nature of engaging key stakeholders in this process has been established, however not summarised (8). Guidelines for competency framework development by Widdett and Hollyforde emphasise that the framework must be relevant to all those who may benefit from its use and meet the needs of a wide range of possible applications (10). To achieve this, the guidelines recommend the process involves a range of stakeholders who will be impacted by the framework. The work of Lundsgaard and colleagues explores how various stakeholders contribute to an understanding of trainee competence and illustrates that incorporating stakeholder perceptions in the development of assessment processes and tools produces a more nuanced and complete conceptualisation of competence (11). However, there has been no examination of the current breadth of stakeholder engagement in competency framework development methodology in the health professions. Understanding if, and how, stakeholders have been involved in competency framework development will identify potential gaps in process. This may further inform guidelines for the development of these frameworks in health professions such that they reflect the work required in order to improve the health and well-being of the population, which is arguably an overarching goal of health care. Educators aim to produce graduates who are cognoscente of population needs at a community and individual level, yet competency frameworks supporting student education may not have considered all aspects of care. Therefore, the aim of this work was to review literature related to competency framework development in health professions, to identify stakeholder engagement in these processes. More specifically, this review aimed to answer the research question: What stakeholders are involved in competency framework development in health professions?

## Methods

### Eligibility Criteria

Eligibility criteria for this review were developed using the PICOS (Participant-Intervention-Comparator-Outcomes-Study design) format (12). Inclusion criteria for the review included original research publications related to methods used to develop competency standards in registered and self-regulating health disciplines including: Aboriginal and Torres Strait Islander Health Practice, Chinese Medicine, Chiropractic, Dental, Medical, Medical Radiation Practice, Nursing and Midwifery, Occupational Therapy, Optometry, Osteopathy, Pharmacy, Physiotherapy, Podiatry, Psychology, Nutrition and Dietetics, Exercise and Sports Science, Audiology and Speech Pathology. Development of specialist competency frameworks within a health profession were included if the results included the process of development of the framework. Specialist frameworks were defined as competency frameworks for a specialist role or those that are specific to a context, population or domain of practise (13). Method papers, such as occupational analysis, were excluded unless they were part of the methodology for the development of competency frameworks.

Publications that did not relate to the methods used to develop or revise competency frameworks, but merely published a framework, were excluded. Research related to competency framework development methodology for a health discipline not included above or pertaining to competency frameworks for preceptors were also excluded.

### Search Strategy

Literature was reviewed systematically to maximise rigour, and the protocol was registered on the PROSPERO International Prospective Register of Systematic Reviews under registration number CRD42019128350: https://www.crd.york.ac.uk/PROSPERO/display_record.php?RecordID=128350. Studies published between 1996 and 2021 were identified by searching MEDLINE, Pubmed, CINAHL Plus with full text, EMBASE and ERIC during March 2019. The search was updated in April 2021 to capture any additional frameworks published up until April 2021. The following key words were used to search:

[(“Aboriginal and Torres Strait Islander Health Practice” OR “chinese medicine” OR chiropract^*^ OR dent^*^ OR “radiation practice” OR nurs^*^ OR midwif^*^ OR “occupational therap^*^” OR optometr^*^ OR osteopath^*^ OR pharmac^*^ OR physiotherap^*^ OR podiatr^*^ OR psycholog^*^ OR “social work^*^” OR dietetic^*^ OR dieti#ian OR “exercise and sports science” OR audiolog^*^ OR “speech patholog^*^” OR “medical education”) AND “competency standard^*^” OR “competency framework” OR “curriculum development”].

The search was limited to English language papers published after 1996, the year of the formal adoption of CanMEDS Roles Framework, a globally recognised physician competency framework (6). This date was deemed relevant as the development of the CanMEDS framework, along with other national frameworks, are critical to the history of competency framework development in health professions, and most standards would have undergone revision since that time. Google Scholar was searched using the same search strategy (automatically sorted by relevance) to identify any publications that did not appear in the database searches. Reference lists of included literature were also hand searched.

Predefined topic and research questions guided data collection, extraction and analysis. The Preferred Reporting Items for Systematic Reviews and Meta-Analyses (PRISMA) statement guided the process of document identification, screening and eligibility assessment (14).

The electronic reference management tool EndNote X9 (Clarivate Analytics, Philadelphia, PA) was used to export and manage references. After the removal of duplicates, one author (BL) independently screened titles and abstracts and selected studies according to the pre-defined inclusion criteria. Risk of bias was managed by ensuring that the full texts of remaining papers were screened in duplicate amongst four researchers (BL, EB, KM, and CP) to identify documents and publications for inclusion. Any discrepancies were discussed in order to reach consensus. The primary author (BL) completed data extraction with oversight from EB.

### Quality Appraisal

To determine quality for review, the full text of each article was assessed independently by the primary researcher (BL), with a sample (20%) checked for accuracy by a second author (EB). Given the variation in research methodologies, the Critical Appraisal Skills Programme (CASP) tool was modified for use, as adapted by Halcomb et al. (15, 16). CASP is the most frequently used tool for quality appraisal and is recommended by the Cochrane Collaboration (17). Articles that did not achieve a “yes” on all items of the checklist were not excluded from the review, but the appraisal was taken into consideration in the overall rigour of the present review. Articles were included if the process of competency framework development process was described with sufficient detail.

### Data Analysis

Data was analysed using techniques of a narrative synthesis, given the heterogeneous nature of the literature (18). This process enables the researcher to construct greater meaning through a process of “re-interpretation” of published findings (19).

For the purposes of this review, we initially defined key concepts and stakeholder groups based on published definitions and author experience (Table 1).

**Table 1:** Definitions of key concepts and stakeholder groups.

**Concept**	**Definition**
Competency framework	A competency framework represents a complete collection of competencies required for effective performance (5)
Practitioners	Practitioners from the profession or specialty of interest
Academics	Individuals who work in education and scholarship at an academic institution
Credentialing or certification organisations	Individuals or a group from a professional body who are engaged in credentialing or certification
Service users	Patients, their family, carers and consumer advocates
Policy makers	Individuals or a group involved in influencing or making policy or who represent Government
Practitioners from other professions	Practitioners from a profession external to the profession of interest (i.e., interprofessional practitioners)
New graduates	New graduate practitioners from the profession or specialty of interest
Education and content experts	External consultants with expertise in curricular design and assessment

## Results

### Search Results

**Figure 1:**
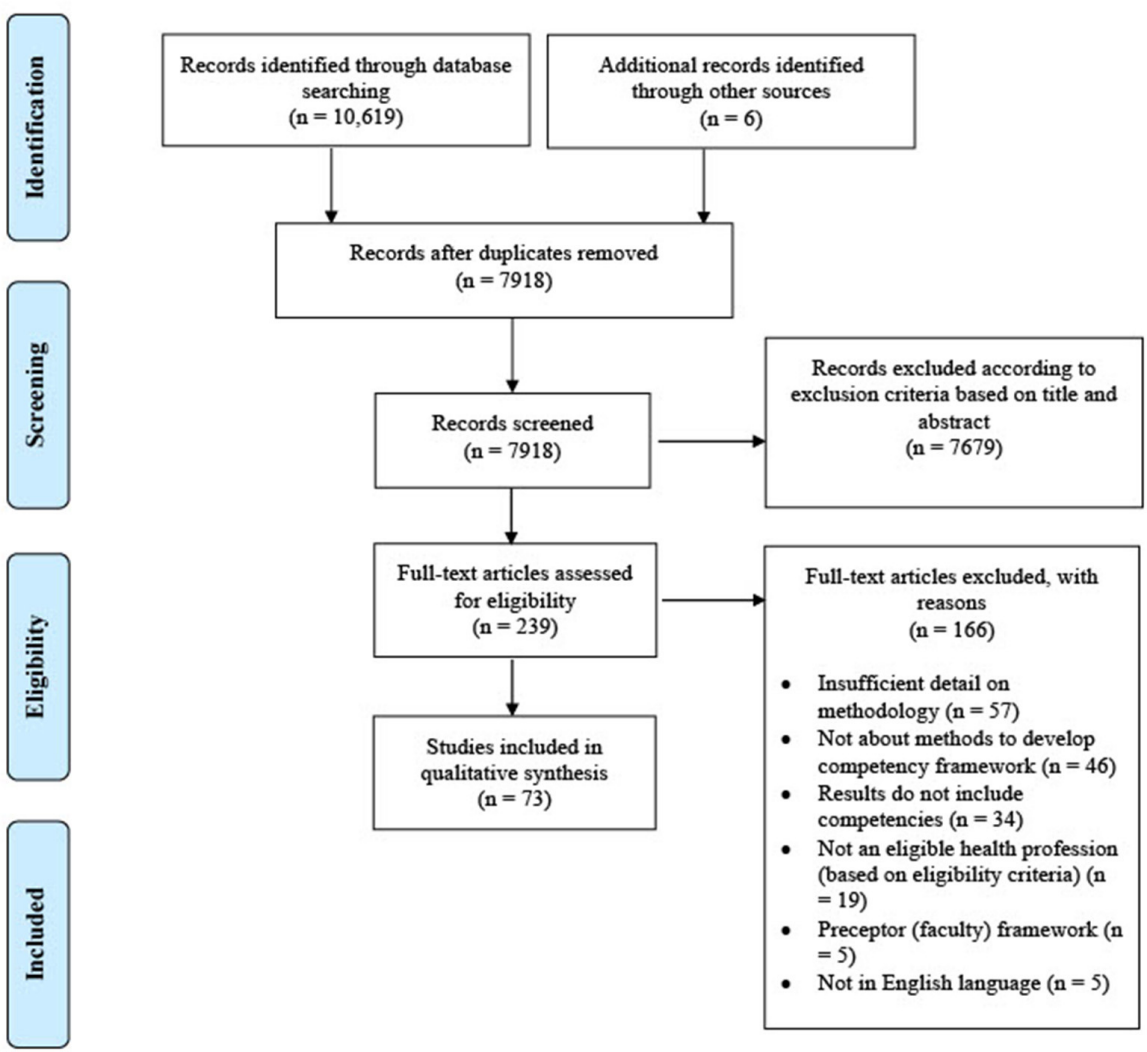
PRISMA flow diagram for identification of studies related to competency framework development methodology in health professions.

The total yield from all databases was 10,625 results, including six articles identified through hand searching of reference lists. This was reduced to 7,918 results after the removal of duplicates. Using the exclusion criteria against title and abstract, a total of 239 full-text publications were assessed for eligibility (Figure 1). Following full-text review, 73 articles were included in the review (Table 2). The reasons for exclusion included insufficient detail on methodology and papers that did not discuss methods to develop competency frameworks.

**Table 2:** Study locations and profession characteristics of included studies (*n* = 73).

**Country**	***n***	**%**
Australia (1, 3–5, 20–36)	22	30%
USA (37–52)	16	22%
UK and Northern Ireland (7, 13, 53–61)	12	16%
Canada (9, 62–67)	7	10%
Africa (68, 69)	2	3%
China (70, 71)	2	3%
Malta (72, 73)	2	3%
South America (74, 75)	2	3%
Italy (76)	1	1%
Japan (77)	1	1%
Korea (78)	1	1%
Kuwait (79)	1	1%
Netherlands (80)	1	1%
Pacific Islands (81)	1	1%
Saudi Arabia (82)	1	1%
Thailand (83)	1	1%
**Profession**	***n***	**%**
Nursing	25	34%
Interdisciplinary	12	16%
Medicine	11	15%
Nutrition and dietetics	5	7%
Optometry	5	7%
Pharmacy	5	7%
Psychology	3	4%
Social work	2	3%
Occupational therapy	1	1%
Midwifery	1	1%
Physiotherapy	1	1%
Radiology	1	1%
Orthotists/prosthetists	1	1%

### Quality Assessment

Quality assessment identified variability in the reporting of study methodology. Rigor of data analysis was the main factor delineating methodological quality between included publications (Supplementary Material). We classified the sampling generously in the first instance, however even with this consideration, the sample size and participant profile were unclear in some publications. In many publications, while significant numbers of stakeholders may have taken part in the competency framework development process, often they were a very homogenous group. Few publications acknowledged study limitations or weaknesses.

### Characteristics of Included Publications

Studies published between 1997 and 2021 were primarily peer-reviewed articles (*n* = 72), with one thesis (*n* = 1). Most publications related to specialty or context-specific competencies (*n* = 55), with 18 pertaining to occupational or professional practise competencies. The majority of studies were from Australia (*n* = 22, 30%), followed by the USA (*n* = 16, 22%), the United Kingdom and Northern Ireland (*n* = 12, 16%), and Canada (*n* = 7, 10%) (Table 2). Nursing competencies accounted for the majority (*n* = 25, 34%), followed by interdisciplinary frameworks (*n* = 12, 16%) and medicine (*n* = 11, 15%) and (Table 2).

### Summary of Results

Studies most frequently engaged four (*n* = 21, 29%) groups of stakeholders in the competency framework development process, albeit this varied considerably from one (*n* = 14, 19%) to seven groups (*n* = 1, 1%). There was considerable variation in the combination of stakeholder groups involved in the competency framework development process. None of the included studies provided a rationale for the number of stakeholder groups engaged in the competency framework development process.

**Figure 2:**
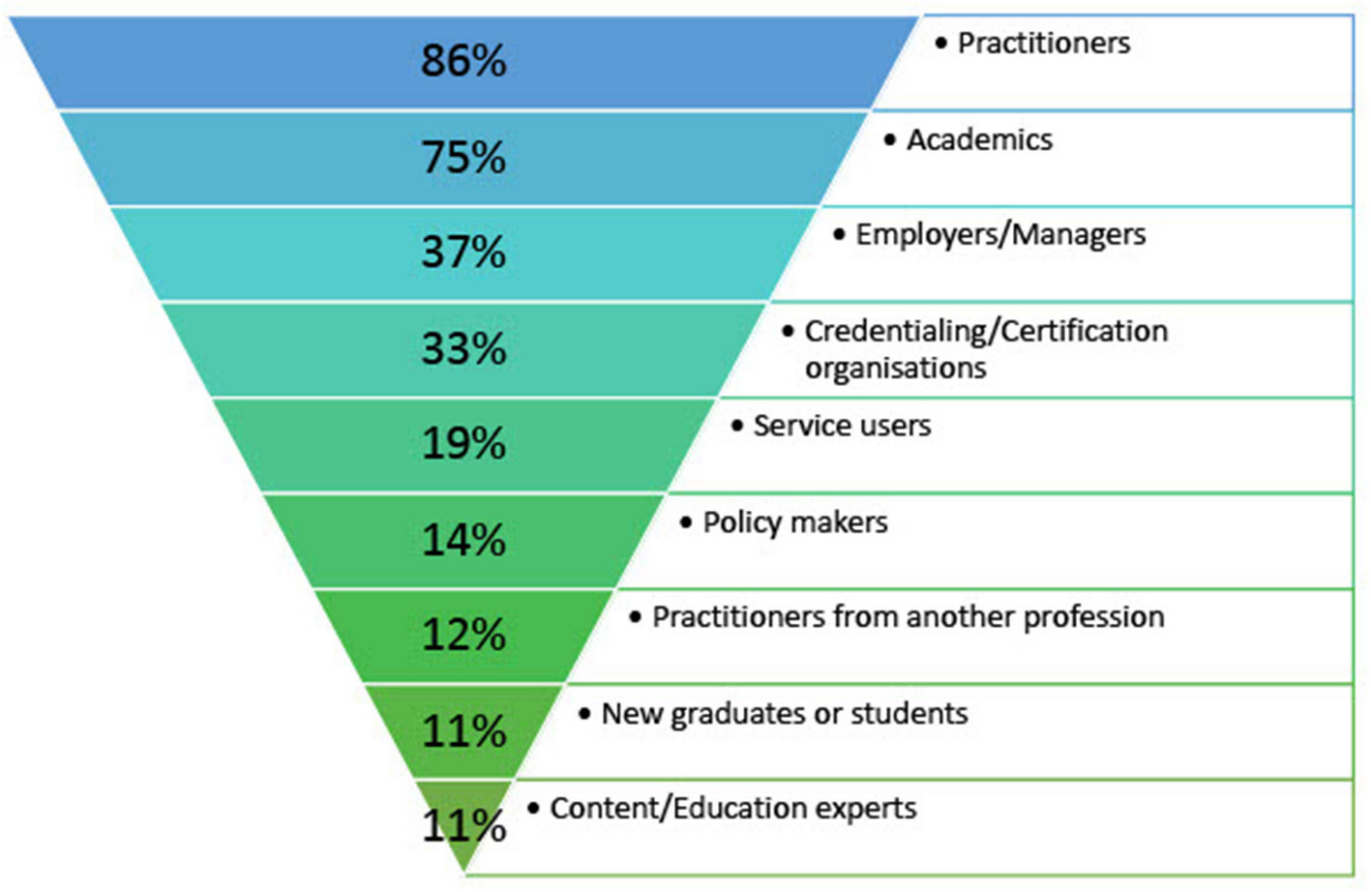
Stakeholder engagement in competency framework development in health professions.

Practitioners and academics were the most frequently engaged stakeholder groups in the development of competency frameworks in health professions outlined in the search strategy (Figure 2). Less than 20% of papers involved service users and only 14% considered the perspective of policy makers in the reported process to develop a competency framework. The least frequently involved stakeholder groups in competency framework development methodology in health professions include practitioners from other professions, new graduates or students and content and/or education experts (Figure 2). Few of the included studies provided a rationale for the stakeholder groups engaged in the competency framework development process in health professions. The findings for each stakeholder group are elaborated on below, with examples to illustrate a critical lack of diversity in stakeholder engagement in competency framework development in health professions.

### Practitioners

Practitioners from the profession were the most frequently engaged stakeholder group in the development of competency frameworks in health professions (Figure 2). Researchers sought practitioner input across a number of methods used to develop a competency framework, in the development of a competency framework, to gather input on an existing framework and to revise, validate or reach consensus on a draught competency framework (Table 3). Practitioners were most frequently involved in studies which reported using the Delphi technique (*n* = 24), interviews, focus groups or a workshop (*n* = 17) as methods to develop competency frameworks. Though there were a small number of papers which reported using values clarification and the nominal group technique as methods to develop a competency framework, all of the papers which utilised these methods engaged with practitioners in the process (Table 3). Practitioners were often selected as Delphi panellists based on clinical expertise; with the assumption they had the necessary experience to be considered “experts” in their field (20, 37, 38, 53, 54, 72). In studies which utilised observation of practise in competency development methodology, there was little effort made to undertake observations in non-clinical settings (settings which do not include one-on-one care), such as roles in public health, management, academia, and policy.

**Table 3:** Stakeholder engagement across reported methods used to develop a competency framework in health professions.

	**Reported methods used to develop competency frameworks**										
	**Total**	**Delphi** **technique**	**Conference** **or workshop**	**Stakeholder** **consultation**	**Interviews**	**Survey**	**Focus** **groups**	**Observation** **of practise**	**Values** **clarification**	**World** **(knowledge) café**	**Nominal group technique**
**Stakeholder group**	***n*** **(%)**										
Practitioners	63 (86%)	24 (96%)	17 (63%)	12 (55%)	17 (94%)	14 (88%)	17 (100%)	6 (100%)	2 (100%)	1 (50%)	2 (100%)
Academics	55 (75%)	24 (96%)	18 (67%)	12 (55%)	4 (22%)	4 (25%)	3 (18%)	1 (17%)	–	2 (100%)	2 (100%)
Employers and/or managers	27 (37%)	8 (29%)	2 (7%)	5 (23%)	4 (22%)	6 (38%)	3 (18%)	1 (17%)	–	–	1 (50%)
Credentialing/certification organisations	24 (33%)	6 (21%)	6 (22%)	13 (59%)	2 (11%)	2 (13%)	1 (6%)	–	–	–	1 (50%)
Service users	14 (19%)	2 (7%)	2 (7%)	5 (23%)	3 (17%)	2 (13%)	3 (18%)	–	2 (100%)	–	1 (50%)
Policy makers	10 (14%)	3 (11%)	3 (11%)	3 (14%)	–	1 (6%)	1 (6%)	1 (17%)	–	–	1 (50%)
Practitioners from other professions	9 (12%)	2 (7%)	–	2 (9%)	3 (17%)	2 (13%)	3 (18%)	–	–	–	–
New graduates or students	8 (11%)	–	2 (7%)	1 (5%)	3 (17%)	1 (6%)	2 (12%)	–	–	–	–
Content/education experts	8 (11%)	1 (4%)	2 (7%)	4 (18%)	1 (6%)	1 (6%)	1 (6%)	–		–	–
Total	73 (100%)	28 (38%)	27 (37%)	22 (30%)	18 (25%)	16 (22%)	17 (23%)	7 (10%)	2 (3%)	2 (3%)	2 (3%)

### Academics

The majority of studies (75%) involved academics in the competency framework development process. Academics participated in the initial development of a competency framework, to solicit input for changes to an existing competency framework and frequently, in the review process. Academics were most often participants in papers which reported using the Delphi technique (*n* = 24), or in a workshop (*n* = 18), and were infrequently engaged in papers which reported utilising observation of practise (*n* = 1). Academics were less likely to be engaged in the process via focus groups and interviews than practitioners (Table 3). In some articles, the development process was limited to the input of a small sample of local, and mostly senior, academics (39–41, 73, 82).

### Employers

Healthcare employers were inconsistently (37%) involved in the competency framework development process. They were most frequently involved as a stakeholder in studies that reported use of the Delphi technique (*n* = 8) or a survey (*n* = 6), though considerably less frequently than practitioners and academics (Table 3). The perspective of healthcare employers or management was sought in the development of an initial competency framework and to revise or validate a preliminary framework. Jie and Wanyi reported including this stakeholder group to provide breadth of perspective on workforce expectation and development (70). The views of employers in the focus group methodology used by Palermo et al. were included to ensure that the competency framework conveyed the professionalism required of the future dietetic workforce (21). Vardanyan et al. reported using interview methodology to explore the perspective of medical co-ordinators on the personal and managerial skills required of pharmacists (74).

### Credentialing and/or Certification Organisation Representatives

Representatives from credentialing and/or certification organisations were involved in the competency framework development process in thirty-three percent (33%) of papers, mostly via a stakeholder consultation process in the end-stages (review and validation) of competency framework development (59%) (13, 42–44, 55, 77). In some studies, the competency framework development process was limited to input from members of the working group, which often comprised of senior representatives from professional bodies engaged in credentialing (40, 56, 75).

### Service Users

Service users such as patients and their family, or consumer advocates were infrequently engaged (19%) in competency framework development, despite many authors claiming a focus on “patient-centred care” (Figure 2). Service users were most frequently engaged in the latter stages of the development process, via a stakeholder consultation (*n* = 5) to review or validate a framework and its application (Table 3). In observation of practise, there were no studies that utilised observation of interactions between clinicians and patients. Authors reported including service users, such as patient representatives and carers, in the development process to ensure that the resulting competency framework encompassed the needs of patients, families and carers (7, 9, 57). Framed as “experts by experience,” Carter et al. note that a focus group with service users helped to shape the content of the admiral nurse competency framework, based on their unique experience and expectations of care (57), though the sample was small (*n* = 5). Cashin et al. (4) state that partnering with service users is fundamental to the development of standards for practise for registered nurses providing person-centred health care. Yet, out of close to 10,000 stakeholders engaged in the redesign of the standards, only seven were patients or consumers. Similarly, Yates et al. (22) circulated a discussion paper outlining key roles and broad competency domains that constitute the specialist breast nursing role in Australia to 60 stakeholders for review, but only one was a representative from a consumer advocacy organisation and no other service users were engaged in the process. In the development of core competencies in cancer genetics for advanced practise oncology nurses, Calzone et al. (38) utilised the Delphi technique with an expert panel, which reportedly included consumer participants (*n* = 9). In this case the “consumers” were graduates of an advanced practise nursing oncology degree, selected to represent “novices in genetics” who are experienced in all aspects of oncology nursing care (38). In some studies, it was not clear how many service users were engaged in the competency framework development process (13, 68, 77). Few papers utilised values clarification as a method in the development of a competency framework, though Davis et al. (13) engaged users of diabetes services in this exercise to develop a competency framework for diabetes nursing. Kirk et al. note that engagement with patient representatives (*n* = 4) *via* a nominal group process validated the application of the competency framework to patient and family needs, and the role of the profession in meeting these needs (7).

### Policy Makers

Policy makers were rarely (14%) engaged as a stakeholder in competency framework development, despite the output of many studies containing competency items related to policy (40, 55, 62, 63). The input of policy makers was sought primarily to review a competency framework, via a workshop or consultation process, and less frequently in the initial construction of such frameworks (Table 3). Lehane et al. purposively included national policy makers (Department of Health) in focus groups in a “creative collaborative process” to develop a competency framework for clinical effectiveness education for health and social care professionals (84).

### Practitioners From Other Professions

Only 12% of papers engaged practitioners from other professions in the process of development, despite competency frameworks containing competency items related to multi-disciplinary care or the ability to work in a team (22, 42, 55). The perspective of practitioners from other professions was most frequently sought using qualitative methods such as focus groups (*n* = 3) and interviews (*n* = 3) in the development of a competency framework (Table 3). Moaveni et al. (64) engaged interprofessional colleagues throughout the framework development process who were able to identify attributes essential for multidisciplinary care. In addition, these same authors reported utilising an interdisciplinary Delphi panel to provide a consensus on what the role of a family practise registered nurse might look like based on multiple perspectives (64). Similarly, Chen et al. interviewed providers from different disciplines, for variety of perspective on clinical experiences and educational needs for the in-patient provider workforce (9).

### New Graduates and Students

Only eight papers (11%) elected to include new graduates or students in the development of competency frameworks, most often using focus group methodology (*n* = 3) (Table 3). Authors reported sampling students or new graduates to capture the evolution of the profession, or to identify competencies required to work in emerging roles (1, 21).

### Content/Education Experts

Content or education experts (also referred to as educationalists) were engaged in 11% of publications, particularly in the end-stages of competency framework development, such as via stakeholder consultation to refine or validate a framework (Table 3). More specifically, authors reported consulting content and education experts to review a competency framework for scope, and to assist with consistency in style, language, and format of the framework. Dressler et al. note that a multiple review process including education experts brought consistency and cohesion to an initially disparate preliminary competency framework (42). Jidkov et al. interviewed stakeholders from a range of expert backgrounds, including educationalists, for diversity in attitude and perspective and to reduce bias in the development of health informatics competencies for postgraduate medical education and training in the UK (58).

## Discussion

There is little existing guidance on stakeholder involvement for competency framework development processes. This study aimed to identify the key stakeholders commonly involved in the competency framework development process in health professions. Our findings build on a previous scoping review of competency framework development (8) by synthesising the evidence and highlighting a lack of diversity in participation of stakeholders in this process. Across methods used to develop competency frameworks, there was a focus on the views of the profession, including practitioners and academics. This is unsurprising, however there is no indication that this is best practise, but rather a reflection on the current status of competency-based education (22). Patients today expect healthcare professionals to not only be experts in their field, but also to work successfully in a team, provide patient-centred care and communicate effectively with patients and colleagues (85, 86). Limiting the conceptualisation of competence to that determined by members of the profession itself may not provide the depth of insight required to capture the complexity of healthcare and address the needs of key stakeholder groups, such as service users (9).

It is well-established that coordinated, team-based health care leads to improvements in health outcomes and patient satisfaction (87). Despite this, only 12% of papers in the present review involved practitioners from other professions in the process of competency framework development. While it is recognised that competency frameworks are limited in terms of describing team competence (88), the application of competencies related to team-based care has been shown to limit re-admissions, improve health outcomes and improve the efficiency and effectiveness of care (87, 89). Poorly structured stakeholder identification and participation, risks the exclusion of valuable perspectives (90). Practitioners from other professions are likely able to identify attributes required to support other key members of the health care team, in order to deliver effective and coordinated health care. Therefore, the participation of interprofessional colleagues in competency framework development may increase the likelihood that the competency framework will encompass standards for team-based health care. Given that coordination of care is a critical component of an effective health system (87), competency framework development should engage other health professions as a stakeholder in order to maximise multidisciplinary understanding and practise (2).

Health 2020 calls for a people-centred health system (91). It is increasingly acknowledged that attention to patients, their family and the community results in improved health outcomes and treatment compliance (92, 93). Here we identified a lack of patient, or service user, perspective as a limitation to competency framework development, with <20% of papers electing to engage patients or consumer advocates in the process. Traditionally, competency frameworks may be heavily reflective of traditional health care settings, which may not have emphasised person-centred care. While person-centred care has become a key aspect of any clinical encounter, the evolution of competency frameworks for health professions appears to lag behind (94). For example, in the development of competency frameworks, only five papers in this review published before 2010 included service users (13, 22, 38, 43). Engagement with patient and family advocacy groups broadens the conceptualisation of competency, particularly given the recent increase in consumer participation in health care (4, 65). Service user participation in health care research may provide unique information about the effectiveness of health care systems including how to improve patient experience and outcomes (95). Patients, and their carers, are intimate with the realities of health system operations and can identify required competencies, from the perspective of those who receive care. Furthermore, consumer involvement, particularly in qualitative methods, such as interviews, focus groups and the nominal group technique, can provide rich insight and reinforce the application of the competency framework to patient and family needs (7). Future competency framework development research should ideally involve a range of stakeholders including service users, such as patients, their family and the wider community.

Previous work has highlighted a need for a wider interpretation of employability, and therefore, of what constitutes professional competence (96). Less than 40% of papers in this review included employers in the competency framework development process. The massification of higher education, a largely stratified and shrinking graduate labour market and an increasing onus on students to improve their own employability skills makes the professional transition to employment a significant challenge (97). Graduate perceptions of employment suggest there is considerable pressure for each individual to develop their own employability relative to others in the post-graduate landscape (97). Without a benchmark, namely, a competency framework, which encompasses professionalism required of the workforce, it is unreasonable to expect an individual to be responsible for their own employability. Collet et al. suggest that competency-based education needs to shift towards enabling graduates to perform in a variety of workplace contexts, and competency centred on organisational purpose (98). Given the complexity of the employment sector described above, the perspective of healthcare employers in the development process is essential in re-shaping the conceptualisation of employability, based on their unique perspective of workforce expectation and demand (21). Ultimately, employability benefits the individual, the workforce, the community and the economy (98). It is therefore in the best interests of all to engage with a variety of stakeholders, including employers in methodologies for competency framework development. Specifically, greater involvement of employers, patients/end users and others in the interdisciplinary team is needed to ensure high quality competency framework development in health professions.

## Strengths and Limitations

This review offers a synthesis of stakeholder engagement in competency framework development in defined health professions and highlights a critical lack of diversity in perspective, however, needs to be considered in the context of its limitations. As healthcare systems are heterogeneous internationally, articles set in other countries, may have used different terms than those used by the authors, resulting in exclusion. Another limitation is that the grey literature included in this review may not be representative of all unpublished studies. Furthermore, the included articles belong to professions across the health care spectrum, where the involvement of stakeholders may vary. There is future scope to directly compare the results of this review to the development of well-established competency frameworks for health professions, such as the CanMEDS Physician Competency Framework (6). Future research should explore shifts in stakeholder engagement in competency framework development in health professions over time and evaluate the downstream implications of a critical lack of diversity in stakeholder engagement on current health care practises.

## Conclusion

A well-defined competency framework serves as a framework to advance the profession, retain practitioners, support growth and development and improve health and safety outcomes for patients. A range of stakeholders may be involved in competency framework development, but to date, there has been a focus only on the views of the profession. Competence in the workforce is a shared responsibility. Therefore, methodologies in future competency framework development should aim to involve a range of stakeholders including other health professions and end-users in competency framework development to match professional education to health service demands.

## Data Availability Statement

The original contributions presented in the study are included in the article/Supplementary Material, further inquiries can be directed to the corresponding author/s.

## Author Contributions

BL, EB, KM, and CP contributed to the design of the review. BL did the literature search, performed data analysis, and drafted the manuscript. EB contributed to quality appraisal. All authors contributed to the literature search, revision of the manuscript, and approval of the final manuscript.

## Conflict of Interest

The authors declare that the research was conducted in the absence of any commercial or financial relationships that could be construed as a potential conflict of interest.

## Publisher's Note

All claims expressed in this article are solely those of the authors and do not necessarily represent those of their affiliated organizations, or those of the publisher, the editors and the reviewers. Any product that may be evaluated in this article, or claim that may be made by its manufacturer, is not guaranteed or endorsed by the publisher.
