# Supplementary material for: Stakeholder Engagement in Competency Framework Development in Health Professions: A Systematic Review
Source: Front Med (Lausanne). 2021 Nov 12;8:759848. doi: 10.3389/fmed.2021.759848 (PMC8632936; doi:10.3389/fmed.2021.759848)
Supplement: Supplementary file 1 [file Table_1.DOCX]

Supplementary Material

**Supplementary Table 1. Quality appraisal**

| **Critical Appraisal Skills Programme (CASP) tool (Halcomb, Stephens et al. 2016, CASP 2018)**   1. Was there a clear statement of the aims of the research? 2. Was the recruitment strategy appropriate to the aims of the research? 3. Was the data collected in a way that addressed the research question? 4. Is the achieved sample size sufficient for the study aims and to warrant conclusions drawn? 5. Was the data analysis sufficiently rigorous? 6. Is there a clear statement of findings? 7. Are the limitations or weaknesses of the study acknowledged? 8. Is the research valuable? |
| --- |

| **Author** | **Year** | **Q1** | **Q2** | **Q3** | **Q4** | **Q5** | **Q6** | **Q7** | **Q8** |
| --- | --- | --- | --- | --- | --- | --- | --- | --- | --- |
| Albarquoni | 2018 | X | X | X | X | X | X | X | X |
| Al-Haqan | 2020 | X | X | X | X | X | X | X | X |
| Ash | 2015 | X | X | X | X | X | X | X | X |
| Attard | 2019a | X | N/A | X | N/A | Unclear | X | Unclear | X |
| Attard | 2019b | X | X | X | X | X | Unclear | X | X |
| Bobonich & Nolen | 2018 | X | Unclear | X | Unclear | | X | Unclear | X |
| Brown | 2012 | X | X | X | Unclear | X | X | X | X |
| Burns | 2014 | X | X | X | Unclear | | X | X | X |
| Calzone | 2002 | X | X | X | X | X | X | X | X |
| Campbell | 2019 | X | Unclear | X | Unclear | | | | X |
| Canning | 2007 | X | X | X | X | X | X | X | X |
| Carrico | 2008 | X | X | X | Unclear | | X | X | X |
| Carrougher | 2018 | X | X | X | X | Unclear | X | X | X |
| Carter | 2018 | X | X | X | X | X | X | Unclear | X |
| Cashin | 2017 | X | X | X | X | X | X | X | X |
| Charles | 2014 | X | X | X | Unclear | | | | X |
| Chen | 2013 | X | X | X | Unclear | X | X | X | X |
| Clark | 2011 | X | X | X | Unclear | | | | X |
| Courtenay | 2018 | X | X | X | Unclear | | X | X | X |
| Curl | 2010 | X | X | X | X | X | Unclear | | X |
| Davis | 2008 | X | X | X | X | Unclear | | | X |
| Dressler | 2006 | X | X | X | Unclear | X | Unclear | | X |
| Dunn | 2000 | X | X | X | X | Unclear | X | X | X |
| Esplen | 2020 | X | X | X | X | X | X | Unclear | X |
| Ford & McIntyre | 2004 | X | X | X | Unclear | X | X | Unclear | X |
| Forsyth | 2018 | X | X | Unclear | | | X | X | X |
| Galbraith | 2017 | X | X | Unclear | | X | Unclear | X | X |
| Gill | 2017 | X | X | Unclear | X | X | X | X | X |
| Greco | 2011 | X | X | X | X | Unclear | X | Unclear | X |
| Halcomb | 2017 | X | X | X | X | Unclear | X | X | X |
| Harding | 2015 | X | X | X | Unclear | | X | Unclear | X |
| Haruta | 2016 | X | X | X | X | Unclear | X | Unclear | X |
| Hay | 2007 | X | X | X | X | X | X | Unclear | X |
| Homer | 2007 | X | X | X | X | X | X | X | X |
| Hoxhaj | 2021 | X | X | X | Unclear | X | Unclear | | X |
| Hughes | 2004 | X | X | X | Unclear | X | X | X | X |
| Hughes | 2015 | X | X | X | X | X | X | X | X |
| Jacono | 2011 | X | X | X | X | Unclear | X | X | X |
| Jenkins & Calzone | 2007 | X | X | X | Unclear | X | X | Unclear | X |
| Jidkov | 2019 | X | Unclear | X | Unclear | | X | X | X |
| Jie & Wanyi | 2018 | X | X | X | X | X | X | Unclear | X |
| Keijser | 2019 | X | X | X | X | X | X | X | X |
| Kiely & Chakman | 1993 | X | X | X | X | X | X | Unclear | X |
| Kiely | 2009 | X | X | X | X | X | X | Unclear | X |
| Kiely | 2000 | X | X | X | X | Unclear | X | Unclear | X |
| Kiely & Slater | 2015 | X | X | X | X | Unclear | X | Unclear | X |
| Kiguli | 2014 | X | X | X | X | Unclear | X | X | X |
| Kirk | 2014 | X | X | X | X | X | X | X | X |
| Kruszewski & Spell | 2018 | X | X | X | Unclear | X | X | X | X |
| Leipzig | 2009 | X | X | X | X | Unclear | X | Unclear | X |
| Lehane | 2020 | X | X | X | X | X | X | X | X |
| Liddell | 2017 | X | X | X | Unclear | X | X | X | X |
| Lim | 2017 | X | X | X | X | X | X | Unclear | X |
| McDaniel | 2014 | X | X | X | Unclear | X | X | Unclear | X |
| Misener | 1997 | X | X | X | X | X | X | X | X |
| Moaveni | 2010 | X | X | X | X | X | X | Unclear | X |
| Palermo | 2016 | X | X | X | X | X | X | X | X |
| Palermo | 2017 | X | X | X | X | X | X | X | X |
| Pastrana | 2016 | X | X | X | X | Unclear | X | Unclear | X |
| Phillips | 2000 | X | X | X | X | X | X | Unclear | X |
| Santy | 2005 | X | X | X | X | Unclear | X | Unclear | X |
| Schofield | 2018 | X | X | X | X | X | X | X | X |
| Shah | 2016 | X | X | X | Unclear | X | X | X | X |
| Suwannaprom | 2020 | X | X | X | X | Unclear | X | X | X |
| Tipson & Turner | 2002 | X | X | X | X | X | X | Unclear | X |
| Vardanyan | 2018 | X | X | X | Unclear | | X | Unclear | X |
| Williams & Hill | 2007 | X | X | X | X | X | X | X | X |
| Wilson | 2014 | X | X | X | Unclear | X | X | Unclear | X |
| Wood | 2009 | X | X | X | Unclear | | X | Unclear | X |
| Yates | 2007 | X | X | X | X | X | X | Unclear | X |
| Zaini | 2011 | X | X | X | Unclear | | X | Unclear | X |
| Zhang | 2019 | X | X | X | X | X | X | X | X |
| Zupanc | 2015 | X | X | X | X | Unclear | X | X | X |

**Supplementary Table 2. Summary data extraction table for included papers**

| **Reference** | **Aim** | **Methods** | **Stakeholders involved** | Description of methods | **Competency framework** |
| --- | --- | --- | --- | --- | --- |
| (Albarqouni, Hoffmann et al. 2018) | To develop a consensus set of core competencies for health professionals in evidence-based practice (EBP). | Literature review, Conference or workshop, Delphi technique, Stakeholder consultation | Practitioners, Academics, Content experts | - A systematic literature review of evidence-based practice (EBP) education was conducted to generate an initial set of competencies for EBP - A 2-round, web-based Delphi survey was used to gain consensus on the essential core EBP competencies for health professionals - The retained competencies were revised based on feedback from participants and arranged into five groups based on level of consensus - The results of the systematic review and Delphi survey were presented at a two-day consensus meeting in order to make final decisions on the inclusion of each competency, wording and description - Consensus was reached on 68 EBP core competencies. The final set of EBP core competencies were grouped into the main EBP domains - To ensure the validity, applicability, utility and clarity of the competencies, the final set of EBP core competencies were sent to 15 EBP experts. | Core competencies for health professionals in evidence-based practice (EBP) |
| (Al-Haqan, Smith et al. 2020) | To develop a foundation competency  framework for pharmacists in Kuwait using the International Pharmaceutical Federation (FIP) Global  Competency Framework (GbCF) in an adopt and adapt approach | Nominal group technique, survey, focus groups | Practitioners, policy makers, academics | - The International Pharmaceutical Federation (FIP) Global Competency Framework was translated from English into Arabic using parallel translation - Two consensus panels involving pharmacists from public and private sector to reach initial consensus on relevance of the FIP competency framework to Kuwait practice - National online survey of pharmacists (n=1483) to identify relevant behaviours and areas of disagreement in the competency framework - Consensus panel with policy and decision makers (n=5) conducted to agree on the list of behaviours to be included in the framework based on the findings from previous phases - Focus group with pharmacists (n=18) to assess the acceptability of the framework as a developmental tool from the end-user’s perspectives. | Kuwait Foundation Competency  Framework (KFCF) |
| (Ash, O’Connor et al. 2015) | To determine the minimum level for entry into the orthotic/prosthetic profession; to develop entry level competency standards for the profession; and to validate the developed entry-level competency standards within the profession nationally, using an evidence-based approach. | Delphi technique, Focus groups, Stakeholder consultation | Practitioners, Academics, Employers and/or managers | - Focus groups were conducted with an expert (n=10) and a recent graduate group (n=8) of Australian orthotist/prosthetists to develop a draft competency framework - A 3-round Delphi survey was used to refine the draft competency framework, resulting in the final draft-competency standards - The final draft competency standards were circulated to 56 members of the Association, who agreed on the key purpose, 6 domains, 18 activities, and 68 performance criteria of the final competency standards. | Competency standards for orthotists/prosthetists in Australia |
| (Attard, Ross et al. 2019) | To identify competencies in spiritual care from an in-depth literature review to develop a spiritual care competency framework for pre-registration nurses and midwives. | Literature review | Academics | - Authors identified cognitive, functional and ethical items in spiritual care for nurses and midwives from a literature review in three areas: spirituality and spiritual care, spiritual care education and curricular contents. - This literature review informs recommendations for spiritual care competencies development, and a theoretical model to underpin a spiritual care competency framework (further developed in Attard et al., 2019b). | A spiritual care competency model |
| (Attard, Ross et al. 2019) | The second of two papers reporting the development of a spiritual care competency framework for pre-registration nurses and midwives. | Focus groups, Delphi technique | Content experts, Practitioners, Service users, Academics, Policy makers | - Five focus groups were held with stakeholders in Malta (chaplains, spiritual leaders, undergraduate nursing and midwifery educators, qualified nurses and midwives, parents and carers, patients and clients) to ensure that aspects of spirituality/spiritual care important to them, but not identified in the literature review, were included in the framework. - The resulting 55 competencies in seven domains formed the Delphi questionnaire which was validated using a two round modified Delphi method involving experts (n=271) in spiritual care from Malta (faculty and clinical nursing and midwifery educators, clinical nurses, policy makers, patients, spiritual leaders, clinical midwives, representatives of clients’ organisations). | A pre-registration spiritual care competency framework |
| (Bobonich and Nolen 2018) | To use a task force of nurse practitioners (NPs) with advanced education and training in dermatology to achieve a consensus for entry-level competencies, followed by validation from an external panel. | Delphi technique, Conference or workshop | Practitioners, Academics, Credentialing and/or certification organisation representatives | - Members of the task force (n=14) completed one pilot and a 2-round Delphi survey to review and assess 91 Delphi items for relevance, specificity, comprehensiveness and whether it was an advanced or entry-level competency - Delphi items that were retained and revised were developed into competency items and organised for review by a validation panel - Validation panel members were invited to a one-day face-to-face meeting to review items again for specificity, relevance and comprehensiveness for dermatology practice. | Competencies for dermatology nurse practitioners |
| (Brown, Gilbert et al. 2012) | To develop a validated pharmacy competency framework for Pacific-Island Countries (PICs). | Survey, Focus groups, Interviews | Practitioners, Employers and/or managers, Practitioners from other professions | - Draft framework was revised using interviews and focus groups in Vanuatu and Papua New Guinea - Wider regional validation of the draft competency framework was undertaken using an online survey tool which required evaluation of the relevance of each competency using a 4-point Likert Scale. | Pharmacy competency framework for the delivery of pharmacy services in Pacific-Island Countries (PICs) |
| (Burns, Lachat et al. 2014) | A two round modified (qualitative) Delphi method was used to develop respiratory protection competencies for occupational and environmental health nurses. | Delphi technique | Practitioners, Academics | - Initial competencies were closely aligned to the nine requirements of the occupational standards for respiratory protection (2011) - A respiratory protection oversight team (n=10) used a modified Delphi method to develop, review and approve initial (draft) respiratory protection competencies developed based on the AAOHN competencies in occupational and environmental health nursing (2007). | Respiratory protection competencies for occupational and environmental health nurses |
| (Calzone, Jenkins et al. 2002) | To determine the competencies expected of all practicing oncology advanced practice nurses (APNs) in the field of cancer genetics. | Delphi technique | Practitioners, Academics, Service users | - 2-round Delphi study with an expert panel (n=37) - Round 1 required open ended responses to identify skills, attitudes and competencies specific to cancer genetics - Round 2 requested ranking of the importance of identified competencies using a 4-point Likert scale. | Core competencies in cancer genetics for advanced practice oncology nurses |
| (Campbell, Harmon et al. 2020) | To revise the 2011 Quad Council Coalition Competencies for Public Health Nursing. | Literature review, Delphi technique, Stakeholder consultation | Practitioners, Academics, Credentialing and/or certification organisation representatives | - A review of the literature included publications and professional nursing competencies in community/public health nursing to inform nursing curricula, guide professional practice and direct public health nursing research and policy development - Task force documented the relationship of the 2011 QCC Competencies to other competency sets identified in the review to identify related and duplicative competencies and gaps in current community and public health nursing competencies - A crosswalk competency review matrix document with the initial findings and recommendations was deployed to the Tier Teams who were asked to review each competency and make recommendations for revision to the assigned tier (delineation), examine each competency for progression from tier to tier and examine the level of the verb used within and across each tier and revise where appropriate - Tier teams then met to examine initial recommendations and rationale and implemented two rounds of the Delphi process to collect and integrate feedback and suggestions - The task force reviewed both rounds of the Delphi process and incorporated feedback into the crosswalk review matrix document - The Task Force convened for a three day retreat to review final recommendations, conduct a secondary review of the literature, define terms, incorporate final recommendations and create the final competency framework for dissemination - The final draft of the QCC Competencies document was submitted to the QCC for consideration and approval. | QCC Competencies for Community/Public Health Nursing |
| (Canning, Rosenberg et al. 2007) | To develop a competency framework that describes the core domains of palliative care nursing. This article explores one key domain of specialist palliative care nursing practice (therapeutic relationships) that was identified as underpinning other domains of practice. | Literature review, Survey, Interviews | Practitioners | - Six core domains were identified in an initial literature review and outlined in an issues paper as the framework for the survey tool - The survey tool comprised a series of items which asked respondents to rate the frequency at which they performed various aspects of their role, using a 4-point Likert Scale - Interviews by telephone with a sub sample of nurses enabled further exploration of the practice examples provided by respondents in the survey - Development of a competency framework based on a synthesis of findings from Phase 1 and 2 as well as comparison of findings to the ANF advanced nurse competency standards (2005) and the ANMC Nurse Practitioner Standards project. The framework was mapped against the PCA standards for providing quality palliative care for all Australians. | A competency framework for specialist palliative care nursing in Australia |
| (Carrico, Rebmann et al. 2008) | To outline basic infection prevention and control (IPC) competencies for hospital-based health care workers, along with specific practice activities, as a means of developing a clear and organized framework as the basis for training and education programs and curricula. | Literature review, Delphi technique | Practitioners | - An initial literature review was conducted to identify existing infection prevention and control competencies for hospital-based health care workers - A Delphi survey was used to revise and reach consensus on the initial competencies | Infection and prevention competencies for hospital based healthcare workers |
| (Carrougher, Hollowed et al. 2018) | To establish a core set of competency standards for burn nursing. | Delphi technique | Practitioners, Academics, Employers and/or managers | - The traditional first round of the Delphi technique (idea generation) was replaced with a request from members of the ABA to submit existing burn nurse competencies - From the review of the competency submissions and in concert with the ANA competency definition, 10 practice domains and 44 competency statements were prepared for the second round - In the second round of the Delphi survey, panellists were asked to respond to 169 questions that included respondent characteristics, ratings for each competency statement for its relevance to the practice using a 10-point Likert scale - Round three included a revised list of competency statements which participants were asked to rate for relevancy using a 10-point Likert Scale - In round four, participants were asked to respond to 18 questions related to use of the competencies and any omissions. | Competency standards for burn nursing |
| (Carter, Bray et al. 2018) | To revise the ANCF-resource for practice (competencies of admiral nurse practice). | Literature review, Survey, Focus groups, Interviews | Practitioners, Service users | - A systematic analysis of existing documentation and relevant articles was conducted to identify which of the original 8 competencies had the greatest relevance to contemporary practice - An online survey was distributed to 130 admiral nurses. The survey asked for information about their job role, how they used the existing framework, anything they felt was missing and how applicable they felt each of the existing competencies was to their practice - Telephone interviews with admiral nurses were conducted to explore survey findings in more detail - A focus group (FG) of people living with dementia and family carers, and a reference group of practitioners was used to develop a competency framework - The competencies were further refined through further focus groups with admiral nurses. | Competencies for admiral nurse practice |
| (Cashin, Heartfield et al. 2017) | To outline the processes that informed the review of the national competency standards for the registered nurse (RN) and development of RN standards for practice. | Literature review, Interviews, Observation of practice, Survey | Practitioners, Academics, Employers and/or managers, Credentialing and/or certification organisation representatives, Service users | - Literature review and critique of existing standards for the RN - One-hour telephone interviews with RNs explored their views of the current RN competency standards and suggestions for revision - Interviews were conducted with 10 health care consumers about their experiences and expectations of RN practice - A total of 44 observations were conducted by a small team of trained observers who are RNs. Using a structured observation tool based on existing standards the following evidence were sought for each standard: Observations of behaviour, thinking out loud and documented evidence - Using an online survey programme two iterations of draft standards were disseminated with the same questions. The first version targeted NMBA stakeholders and the second iteration was disseminated as an open public consultation - For validation, a final round of 35 observations was conducted in a range of clinical and non-clinical settings in each state and territory. | Australian registered nurse (RN) standards for practice |
| (Charles, Triscott et al. 2014) | To describe the development and implementation of incremental core competencies for Postgraduate Year (PGY)-I Integrated Geriatrics Family Medicine, PGY-II Geriatrics Rotation Family Medicine, and PGY-III Enhanced Skills for care of the elderly (COE) Diploma residents at a Canadian University. | Literature review, Conference or workshop | Academics | - A core competency working group was established using interested and experienced members of the COE residency program committee - The 20 core competencies for medical students, developed by the Medical Education Committee of the Canadian Geriatrics Society were selected by the working group as a baseline. An additional seven competencies were received from the national health care of the elderly committee on core competencies at the PGYI-II level. Each working group member then worked on competencies in a number of domains, developing core competencies expected at the PGYI/II and PGY III level of training - Draft competencies were then circulated to each individual member of the working group who was asked to independently review the core competencies and indicate those that were appropriate and if so, then for which level - Individual members then anonymously submitted this information to the chair of the working group - Statements that did not reach consensus were identified and discussed formally with all members of the working group. | Geriatric core competencies for family medicine |
| (Chen, Krupa et al. 2013) | To assess in-patient providers’ recovery educational needs and identify the most salient components of recovery competencies. | Literature review, Interviews | Practitioners, Academics, Service users, Practitioners from other professions | - Literature review related to current practice, including the therapeutic environment and culture of the setting, existing competencies for providers working in this context and the challenges and barriers toward developing these competencies and in the provision of safe, effective services - Semi-structured, face-to-face key informant interviews conducted to identify the most salient components of recovery competencies in inpatient psychiatric settings. Consumers and family participants were also asked about their expectations of providers. All participants were asked to discuss challenges providers may face in demonstrating these competencies. Codes from interview data were clustered into groups and findings from the literature review were then integrated into the groups of codes - A preliminary conceptual framework was developed to organise the first category of themes and explain their relationships. Remaining transcripts were analysed, and new findings added to the conceptual framework. | Recovery competencies for in-patient mental health providers working with people with serious mental illness |
| (Clark, Calvillo et al. 2011) | To report on the development of the American Association of Colleges of Nursing (AACN) cultural competencies and toolkit for master’s and doctoral nursing education. | Literature review, Conference or workshop | Practitioners | - A literature review on how nursing and other health-related disciplines conceptualized and designed cultural competency programs was synthesized - The AACN advisory group formulated six initial core cultural competencies which were initially reviewed, revised and then endorsed by the AACN board of directors. | Cultural competencies for graduate nursing education |
| (Courtenay, Lim et al. 2018) | To provide UK national consensus on a common set of antimicrobial stewardship (AMS) competencies appropriate for undergraduate healthcare professional education. | Delphi technique | Practitioners, Academics, Employers and/or managers | - A modified Delphi approach comprising two online surveys delivered to a UK national panel of 21 individuals reflecting expertise in prescribing and medicines management - The first Delphi round consisted of pre-defined competency statements and associated descriptors. Participants were asked to use a 6-point Likert Scale to rate the importance of each descriptor. At the end of each domain, an open-ended questionnaire invited panel members to provide their interpretation and feedback and to identify any additional descriptors they thought were missing - Only panel members who completed round one were invited to participate in round two. Only descriptors for which there was a lack of agreement, descriptors that were amended in the light of qualitative feedback and additional descriptors identified by panel members were included in the round two questionnaire. | Antimicrobial stewardship (AMS) competencies for UK undergraduate healthcare professional education |
| (Curl, Tompkins et al. 2010) | To gain support for a list of competencies needed by social workers who specialize in gerontological practice. | Literature review, Survey | Practitioners, Academics, Employers and/or managers | - A literature review of social work, gerontology and interdisciplinary aging practice literature, particularly existing gerontological competencies and how they were developed - A copy of the preliminary competencies was sent to 7 nationally recognised social work gerontology expert consultants for review. | Gerontological social work competencies |
| (Davis, Turner et al. 2008) | To describe the development of an integrated career and competency framework for diabetes nursing. | Values clarification, Stakeholder consultation, Workshop or conference | Practitioners, Credentialing and/or certification organisation representatives, Service users | - A purposive sample of nurses representing all sectors and grades of staff involved in diabetes care was invited to workshops to undertake a values clarification exercise. Content analysis was performed to identify themes - Further workshops identified areas of specialist practice and competencies were developed and refined in a series of consultations - To ensure transparency and maintain group ownership of the work, an amended draft was sent to workshop attendees for comment, revision and approval - Another revised draft was then sent out to over 200 stakeholders for review. | A competency framework for diabetes nursing |
| (Dressler, Pistoria et al. 2006) | To develop core competencies for hospital medicine. | Survey, Stakeholder Consultation | Practitioners, Academics, Credentialing and/or certification organisation representatives, Content experts | - The task force compiled an initial list of competencies in three domains - The task force developed a survey to obtain feedback on the initial topic list from the editorial board, contributors, relevant committees and external reviewers of medical professional and academic organisations. This process culminated a core competencies document which is divided into three sections. | Core competencies in hospital medicine |
| (Dunn, Lawson et al. 2000) | To develop competency standards for critical care nurse specialists and articulate the differences between entry-to-practice standards and the advanced practice of specialist critical care nurses. | Interviews, Observation of practice, Conference or workshop | Practitioners, Academics | - Observation of critical care nursing practice and follow-up interviews with observed nurses was undertaken at 57 hospitals - Further validation of the interpretations from observation and interview data was carried out at a national workshop until consensus was achieved - Following the workshop, the draft was returned to data collectors to discern if the standards reflect what was observed in clinical practice. Comments and suggestions were incorporated to produce the final document. | Competency standards for specialist critical care nurses |
| (Esplen, Hunter et al. 2020) | To develop a competency  framework with relevance across regulated health professionals involved in cancer care. | Literature review, Focus groups, Interviews, Survey, Delphi technique | Practitioners, academics | - Focus groups and interviews (n=51) with practitioners (interdisciplinary) were conducted to collect data on local interest in continuing education opportunities and cancer-related topics considered most relevant and necessary to support practice. - Survey of nurses to solicit their views in relation to educational preparation and current need. - Scoping review of national and international guideline and standards for cancer care, and relevant competencies was undertaken. - From multiple sources, the working group identified core competency domains. - A modified Delphi technique with three rounds was used to build consensus on specific competencies and the level of the competency. - Items which achieved 80% or greater agreement were included. | The Interprofessional de Souza Specialist Framework of Competencies |
| (Ford and McIntyre 2004) | To develop baseline competency standards for occupational therapy (OT) assistants. | Literature review, Focus groups, Interviews, Observation of practice, Stakeholder Consultation | Practitioners, Practitioners from other professions, Employers and/or managers | - A preliminary literature review of research relating to OT assistant and standards of practice. - Focus groups with OTs and OT assistants were used to identify draft competencies. Discussion identified attributes of OT assistants, tasks they commonly perform and variables in the range of work roles. - Draft competencies were validated through structured observation of 8 OT assistants in the workplace. - Critical incident interviews were conducted with 4 OT assistants and 4 OTs (n=8). Information gathered was analysed and resulted in subsequent refinement of the draft competencies - The penultimate draft was reviewed by the manager of OT services, 6 senior OTs, 4 nurse unit managers and the participants of the focus group, structured observation and critical incident interviews. | Competency standards for occupational therapy (OT) assistants |
| (Forsyth, Warren et al. 2018) | To provide an expert consensus on the minimum competencies necessary for clinical pharmacists to deliver appropriate care to patients with heart failure. | Literature review, Conference or workshop | Practitioners, Academics, Credentialing and/or certification organisation representatives | - A literature review of existing pharmacy competency frameworks in other specialities and previous health failure curricula was conducted to identify existing heart failure specific frameworks and existing frameworks for advanced pharmacy practice, both generalist and specialist - Three authors developed the initial framework. The RPS framework was adapted to define baseline competencies of all pharmacists. Contemporary international heart failure guidelines and previous systematic reviews and meta-analyses were used to inform the initial framework. In areas where there was no evidence expert opinion was applied. | A competency framework for clinical pharmacists and heart failure |
| (Galbraith, Ward et al. 2017) | To synthesise from empirical research a real-world evidence-based medicine (EBM) framework for general practice. | Literature review, Delphi technique | Practitioners, Academics | - Two sets of themes describing the meaning of EBM in general practice were synthesised to generate a competency framework - One set of themes was derived from a mixed-methods systematic review of the literature; the other set was derived from the further development of those themes using a two-round Delphi process among a panel of EBM and general practice experts (n=10). | An evidence-based medicine competency framework for general practice |
| (Gill, Kendrick et al. 2017) | To revise the ACCCN Competency Standards for Specialist Critical Care Nurses to ensure they continue to meet the needs of critical care nurses and reflect current practice. | Focus groups, Delphi technique | Practitioners, Academics, Employers and/or managers | - Twelve focus groups (n=79) were conducted with specialist critical care nurses. The standards were revised based on the main themes - A national panel of critical care nurses responded to a three round Delphi-survey, using a 7-point Likert-type scale to indicate their level of agreement with the revised standards. | Australian practice standards for specialist critical care nurses |
| (Greco, Tinley et al. 2011) | To create a consensus document identifying the genetic/genomic competencies essential for nurses prepared at the graduate level. | Literature review, Delphi technique | Academics, Credentialing and/or certification organisation representatives | - A systematic literature review of articles published in the US as well as well as health professional websites and references from relevant articles identified competencies for a first draft - 31 representatives from stakeholder organisations were invited to participate in a consensus panel for the draft competencies. The panel reviewed the relevance and comprehensiveness of each draft competency. Each competency item that failed to receive 100% agreement on all 3 elements was revised or deleted as appropriate - A second online questionnaire was created to capture votes and comments on the 24 competencies that were deleted or revised. | Genetic and genomic competencies for graduate nurses |
| (Halcomb, Stephens et al. 2017) | To explore the current role of general practice nurses and the scope of nursing practice to inform the development of national professional practice standards for Australian general practice (GP) nurses. | Focus groups, Survey, Conference or workshop, Stakeholder Consultation | Practitioners, Academics | - Focus groups with registered and enrolled nurses currently working in Australian GP using open-ended questions and changing the content of groups over time - Two online surveys of registered and enrolled nurses currently working in GP. The first survey sought to collect feedback on the previous version of the competency standards and identify areas that were either not address or required further development. The second online survey sought to validate the revised standards. | Professional practice standards for Australian general practice (GP) nursing |
| (Harding, Prescott et al. 2015) | To develop a clinical education framework that included an agreed competency standard and credentialing process to support advanced musculoskeletal physiotherapy (AMP) roles. | Literature review, Focus groups | Practitioners | - A scoping review was conducted to determine if any education, training and competency resources were currently available for AMP roles - Two focus groups were conducted with physiotherapists working in AMP roles. Discussion focused on key attributes requires for working in AMP roles and education and training for AMP roles. These attributes were grouped into broad domains that were also identified by participants - All participants were invited to a third follow up focus group to discuss the outcomes and verify the framework. | A competency framework to support advanced musculoskeletal physiotherapy (AMP) roles |
| (Haruta, Sakai et al. 2016) | To identify a set of competencies to prepare Japanese students and healthcare practitioners for collaborative practice. | Literature review, World Café method, Conference or workshop, Stakeholder consultation | Practitioners, Academics, Credentialing and/or certification organisation representatives, Service Users | - In order to confirm the development process, a review and comparison of existing international interprofessional competency frameworks was conducted - Two academic meetings were held (n=21 participants) using the World Café method in which they developed an extensive pool of comments and opinions - A workshop was held simultaneously (n=30) - A core project team reviewed data from thematic analysis of the meetings. The team developed prototype international competency domains from this data - Representatives (n=7) from professional organisations in medicine, nursing, pharmacy, physical therapy, OT, nutrition and social welfare were invited to confirm understanding of the concept and transferability at a conference, by discussing the draft proposal - An open symposium with stakeholders (n=105), including higher education faculty and staff, health professionals, patients and the public was conducted to validate the competencies - A meeting with representatives from professional organisations in the field of healthcare and social welfare was held to reach a final agreement on the interprofessional competency framework. | An inter-professional competency framework for Japanese students and healthcare professionals |
| (Hay, Campbell et al. 2007) | To develop a specialty-specific set of competencies required by a GP with a special interest (GpwSI) in working within the field of musculoskeletal/rheumatology practice. | Literature review, Delphi technique, Conference or workshop | Practitioners, Academics, Practitioners from other professions | - A comprehensive review of existing relevant literature and curricula was carried out and the results collated to produce a list of proposed competencies - A Delphi process was used to reach consensus on the proposed competencies - Participants in the first round (n=16) were asked to score individual items on their relevance using a 4-point Likert scale. All items scoring 3 or 4 were excluded for the next round - Participants were asked to rescore each item in the second round using the same scale and indicate depth of knowledge required for each competency - Two workshops were held with representatives from musculoskeletal and rheumatology interest groups and those with an interest in GP education. The output from the workshop was formatted according to an educational framework of 12 domains common to other work with practitioners with a special interest - A 6-month consultation period resulted in some editorial changes to the style of the final document | A competency framework for GPs with a special interest in working within the field of musculoskeletal/rheumatology practice |
| (Homer, Passant et al. 2007) | To develop and validate national competency standards for midwives in Australia. | Literature review, Survey, Conference or workshop, Observation of practice, Interviews | Practitioners, Academics, Employers and/or managers, New graduates or students | - A draft document was compiled from an initial literature review - The draft document was used in eight workshops with 153 midwives, students and policy makers - A survey and written submissions were used to further review the competency framework - Direct observation of practice in a range of settings ensured validation of the competencies. | National competency standards for midwives in Australia |
| (Hoxhaj, Tognetto et al. 2021) | To identify the core competencies in cancer genomics for non-genetic healthcare professionals (physicians and nurses). | Literature review, Delphi technique | Practitioners | - Literature review of competencies for non-genetic health professionals in cancer genomics. - Web-based, two-round modified Delphi survey conducted with specialists (n=8) in genetics, genomics, oncology and medical specialists to define, through consensus (70% agreement level), a set of core competencies for curricula. - Delphi panellists could also propose additional competencies based on their own experience. | Core competencies in cancer genomics for physicians; Core competencies in cancer genomics for nurses |
| (Hughes 2004) | To assess the level of consensus amongst an international panel of public health nutrition leaders regarding the essential competencies required for effective public health nutrition practice. | Literature review, Delphi technique | Practitioners, Academics | - A literature review was conducted as a prelude to the expert panel survey and was used to identify common competency units from the fields of public health, health promotion, nutrition and dietetics, and health education - A panel of 20 public health nutrition experts from seven countries in the European Union, the USA and Australia were invited to participate in a modified Delphi study involving three rounds of questionnaires. | A competency framework for effective public health nutrition practice |
| (Hughes, Begley et al. 2015) | To assess consensus among public health nutrition (PHN) workforce development stakeholders on the competencies required for effective PHN practice in the Australian workforce context. | Literature Review, Delphi technique | Practitioners, Academics, Employers and/or managers | - A modified, 2-round Delphi survey with an expert panel of 33 Australian PHN workforce development stakeholders was used to reach consensus on competencies required for effective PHN practice in the Australian workforce context - Surveys tested panellist ratings (essential, useful, irrelevant) of a listing of 143 competency elements derived from the literature and existing competency standards, across rounds, with feedback between rounds. | A competency framework for effective public health nutrition practice in Australia |
| (Jacono, Young et al. 2011) | To present consensus based, national nursing competencies in palliative end-of- life care (PEOLC), developed in a Canadian Association Schools of Nursing (CASN) project funded by Health Canada. | Literature review, Survey, Conference or workshop | Practitioners, Academics, Employers and/or managers, Credentialing and/or certification organisation representatives, Practitioners from other professions, New graduates or students, Content experts | - A working group of the CASN task force reviewed nursing literature on palliative and end-of-life care to generate a draft set of competencies - A web-based survey was sent to ninety-one CASN member schools containing categorical (nominal) response choice questions. Each question included the option of adding comments or concerns about the competencies - A national symposium was held with 32 nurse educators, nursing students and representatives from nursing organisations and government, all with expertise in PEOLC. Consensus-based comments on the competencies were collated and used to revise the competencies following the symposium - The revised set of competencies were sent to CASN member schools for feedback - Finally, 95 content experts were asked to review the competencies using an online survey tool - Four experts and the task force were asked to validate that the refined competencies continued to reflect competencies that emerged in the consensus building phase. | Palliative care competencies for Canadian nurses |
| (Jenkins and Calzone 2007) | To reach consensus on essential (core) genetic and genomic competencies relevant to the entire US nursing profession. | Literature review, Conference or workshop, Stakeholder consultation | Practitioners, Academics, Credentialing and/or certification organisation representatives, Service users | - A steering committee identified, reviewed, analysed and compared competencies recommended in published and peer-reviewed literature - A writing group consisting of members of the steering committee was established to analyse and synthesise these documents to identify fundamental genetic and genomic competencies applicable for all registered nurses - Draft competencies were presented to nurse representatives and posted by the American Nurses Association (ANA) for public comment - An in-person meeting with key stakeholders from the nursing community was conducted to reach consensus on the competencies. | Nursing competencies for genetics and genomics |
| (Jidkov, Alexander et al. 2019) | To assess health informatics (HI) training in UK postgraduate medical education, across all specialties, against international standards in the context of UK digital health initiatives (eg, Health Data Research UK, National Health Service Digital Academy and Global Digital Exemplars). | Literature review, Interviews | Practitioners, Practitioners from another profession, Academics, New Graduates | - A scoping review was undertaken to identify existing HI standards for doctors - A snowball technique was used to identify additional relevant references - Authors used the 2000 and 2010 International Medical Informatics Association Recommendations for Biomedical and HI Education as a framework for mapping HI competencies, to which additional unidentified competencies were iteratively added - Content analysis of published postgraduate medical curricula identified from, or via the GMC website, was conducted to identify HI in postgraduate training - Two experts were interviewed by telephone and five experts had face-to-face interviews - Experts were selected via convenience sampling, and others using a snowball technique - Experts included physicians, curriculum developers, educationalists and a data scientist to provide a range of attitudes and knowledge bases and avoid bias - Experts were consulted regarding outputs of the scoping review and curricular content analysis and invited to consider universal HI competencies for all doctors. | Universal Health Informatics Competencies for Doctors. |
| (Jie and Wanyi 2018) | To derive a competency framework from the perspective of Chinese experts in social work, and to reveal the reasons for these particular choices. | Literature review, Delphi technique | Academics, Employers and/or managers | - Working in reference to professional competencies that have been identified by the USA, England and Hong Kong, twenty Chinese academics, fourteen Directors/Deputy Directors and fifteen senior practitioners individually chose those competencies they perceived as important to generate a draft framework - The Fuzzy Delphi method was used to reach consensus on competencies for social workers in China. | Competencies for social workers in China |
| (Keijser, Handgraaf et al. 2019) | To develop a Dutch medical leadership (ML) competency framework and to completely to known approaches of developing such frameworks. | Literature review, Interviews, Focus Groups, Survey | Practitioners, Academics, Practitioners from another profession, New Graduates or students, Policy makers | - Literature review of scientific and grey literature to provide context for data interpretation and to ensure optimal reflection of the relevant needs in the Dutch healthcare system. - Axial coding was used to categorise coded fragments into competency domains which reflect themes of medical leadership. - Semi-structured interviews (n=31) with medical professionals and non-medical professionals from other professions, healthcare management and patient advocacy services. - Three researchers developed an initial coding list of 47 labels by independently screening a randomly selected sample of three transcripts. The list was tested by individually coding a fourth randomly selected transcript, revealing a 90% inter-coder correspondence. - Six researchers then independently coded all the remaining transcripts in pairs until saturation was reached. - All the included literature and transcripts were synthesised and coded to provide a definition of medical leadership. - The DML framework was validated through an online survey (and three focus group discussions. - Finally, during a final consensus session, the core group members constructed a graphical representation of the relationship between the domains and overarching dimensions: ‘me’, ‘others’ and ‘society’. | Dutch Medical Leadership Framework v1.0 |
| (Kiely and Chakman 1993) | To determine competency standards for entry-level to the profession of optometry in Australia. | Conference or workshop, Interviews, Observation of practice, Stakeholder consultation | Practitioners, Academics, Credentialing and/or certification organisation representatives, New graduates or students | - A modified functional analysis approach was used to explore the key purpose of jobs and roles in the whole occupational sector - Two workshops were conducted using the same participants on both occasions - A draft framework was developed and sent to the steering committee and members of the workshops for comment - Nineteen graduates were interviewed using the critical incident technique. Critical incident questionnaires were also sent to all other graduates from that year. Two researchers read these interviews and interpreted the data to refine and validate the results of other techniques used and to determine if there were any competencies missing - Three graduates and 9 practitioners were observed in their practices. The research officer attended all consultations provided by the practitioners, recorded details of all tests performed and made note of attitudes, skills and knowledge required and exhibited by the optometrist. The optometrists were also invited to comment on the draft standards - To enable practitioners not directly involved in the workshops and interviews to make contributions to the drafts of the competency standards, articles were included in the monthly AOA newsletter to allow a consultative/feedback mechanism - Final workshop comprising members of the steering committee considered the comments made to the draft and a telephone conference of the steering committee was subsequently held to make final amendments. | Competency standards for entry-level into the profession of optometry in Australia |
| (Kiely 2009) | To revise the universal (entry-level) and therapeutic competency standards for the profession of optometry in Australia. | Literature review, Stakeholder consultation, Conference or workshop | Practitioners, Academics, Credentialing and certification organisation representatives | - A literature review was conducted to identify which standards similar to competency standards were in place for optometry globally and for other health professions in Australia, and to determine whether there were any areas addressed in these standards not contained in the 2000 Australian entry-level and therapeutic competencies - The 2000 document was circulated to over 80 optometrists in Australia and members of optometrists’ registration boards for suggestions about how the standards could be altered to reflect expectations for entry-level to the profession of optometry and requirements for therapeutic endorsement. The resulting comments were incorporated into a master document - The master document was analysed and refined at a workshop comprising 12 optometrists. Recommendations from the workshop were incorporated into a second draft and returned to participants for further comment - The standards were then sent to state divisions of Optometrists Association Australia for further comment and refinement. | Entry-level and therapeutic competency standards for the profession of optometry in Australia |
| (Kiely, Chakman et al. 2000) | To develop therapeutic competency standards for the profession of optometry in Australia. | Literature review, Stakeholder consultation | Practitioners, Academics, Credentialing and/or certification organisation representatives | - An initial draft of therapeutic CS was developed at the Optometrists Association Australia using information obtained through a previous literature search, analysis of requirements for therapeutic use in other jurisdictions, analysis of existing optometric therapeutic courses and consultation with experts in the field - Draft competencies were distributed for comment to representatives from the optometry schools and registration boards in ANZ, state divisions of OAA, NZ Association of Optometrists and up to 30 members of the profession with particular expertise and interest in the field - The second draft was circulated to the same groups and individuals for a second round of comments. | Therapeutic competency standards for the profession of optometry in Australia |
| (Kiely and Slater 2015) | To include therapeutic competency standards in the entry-level competencies and to review and revise entry-level competencies for the profession of optometry in Australia. | Literature review, Conference or workshop, Stakeholder consultation, Survey | Practitioners, Academics, Employers and/or managers, Credentialing and/or certification organisation representatives | - Literature review to determine existing competency standards for optometry and for other health professions in Australia. Under the guidance of the steering group, amendments were made to the 2008 version of the standards on the basis of the literature review and consideration of international practice of optometry to develop a draft competency document - This document was considered at a series of four workshops with practising optometrists with each workshop addressing different units of competency. Recommendations from the workshops were collated into a further draft - All members of the then Optometrists Association Australia for whom email addresses were held were invited to comment on the draft competency standards. In addition, specific invitations were made to key stakeholder organisations - Comments from submissions were considered in consultation with the steering committee and incorporated into a further draft - State Divisions of Optometrists Association Australia were then invited to comment on the draft. Following incorporation of the minor suggestions received from State divisions, the revised competency standards were presented to the National Board of the then Optometrists Association Australia for adoption. | Entry-level competency standards for the profession of optometry in Australia |
| (Kiguli, Mubuuke et al. 2014) | To define the required competencies of graduating doctors in Uganda and implement competency‐based medical education (CBME). | Literature review, Conference or workshop | Practitioners, Academics, Employers and/or managers, Credentialing and/or certification organisation representatives, Service users, New graduates or students | - Faculty and leaders from universities, as well as clinicians practicing in the teaching hospitals of the universities participated in two workshops to identify Uganda’s priority health needs - Three face-to-face workshops were conducted with the same participants involved in the identification of Uganda health priorities through earlier workshops. These workshops involved discussing the concepts of CBE led by faculty with expertise in medical education followed by small group work to develop a draft competency framework - The draft was circulated to leaders of individual consortia institutions to get feedback and input through workshops and meetings. | Competencies for graduating doctors in Uganda |
| (Kirk, Tonkin et al. 2014) | To report a review of a genetics education framework using a consensus approach to agree on a contemporary and comprehensive revised framework. | Nominal group technique | Practitioners, Academics, Employers and/or managers, Credentialing and/or certification organisation representatives, Service users | - A meeting was conducted involving stakeholders in UK nursing education, practice and management, including patient representatives (n = 30). A consensus approach was used to solicit participants’ views on the individual/family needs identified from real-life stories of people affected by genetic conditions and the nurses’ knowledge, skills and attitudes needed to meet those needs. Five groups considered the stories in iterative rounds, reviewing comments from previous groups. Omissions and deficiencies were identified by mapping resulting themes to the original framework. Anonymous voting captured views - Educators at a second meeting developed learning outcomes for the final framework. | Genetics and genomics competency framework for nursing education in the UK |
| (Kruszewski and Spell 2018) | To identify a core set of quality improvement and patient safety (QI/PS) items to be taught during medical school, residency, and independent practice, with specificity to guide curriculum development at each level. | Delphi technique | Academics | - A panel of 12 QI leaders and educators with backgrounds in internal medicine from 10 academic institutions participated in consensus development using a modified Delphi technique. Three rounds of anonymous surveys were conducted, followed by a teleconference and then a fourth survey round, until consensus regarding the relevance of candidate items was reached. | Quality improvement and patient safety (QI/PS) competencies in medicine |
| (Leipzig, Granville et al. 2009) | To identify and define the minimum geriatrics-specific competencies needed by a new intern to adequately care for older adults. | Conference or workshop, Survey | Practitioners, Academics, Employers and/or managers, Credentialing and/or certification organisation representatives | - A draft competency framework was generated by geriatricians at a working conference, by identifying measurable performance subtasks associated with accepted standards of evidence-based geriatric care, patient safety, and “do no harm” within the first-year resident’s expected scope of practice - Draft competencies were then assessed for content validity by key stakeholders and informants at a consensus conference - The alpha competency draft and content validity survey results served as the focus for a 2.5-day working conference attended by 98 people who responded to a combination of targeted invitations and general announcements about the conference. | Geriatrics competencies for graduating medical students |
| (Lehane, Agreli et al. 2020) | To develop a competency framework for education in evidence-based practice and clinical effectiveness to ensure responsiveness of education standards and curricula of healthcare professionals in this area. | Literature review, Focus groups | Academics, Credentialing and/or certification organisation representatives, Service users, Policy makers | - Review of national and international reports, professional guidance documents and empirical literature pertaining to clinical effectiveness education to develop a preliminary competency framework. - Thirteen focus groups with stakeholders (n=45) to elucidate participants’ perspectives on clinical effectiveness education competencies for healthcare professionals and examine proposed competencies of clinical effectiveness education for relevancy, clarity and comprehensiveness. | Competency framework for clinical effectiveness education |
| (Liddell, Allan et al. 2017) | This study explored the therapeutic competencies required to deliver compassion focused therapy (CFT) and organized these into a coherent framework. | Interviews, Delphi technique | Practitioners, Academics | - The first round of data collection involved interviews with 12 experts in CFT. Data were analysed using template analysis to generate a draft competency framework. The main competencies were used to create a survey for rounds two and three involving CFT experts and practitioners. - Data collected from the two survey rounds were used to refine the competencies. | The compassion focused therapy (CFT) competency framework |
| (Lim, Lee et al. 2017) | This study aims to clarify the job competency of Korean radiological technologists (RTs). | Literature review, Survey | Practitioners | - A task force (n=11) of professional RTs examined existing competency requirements for RTs in other countries to identify preliminary competencies - A survey was developed and distributed to RT professors, managers in large healthcare organisations and general RTs to evaluate the validity of the preliminary competencies. The survey required respondents to assess items using a 5-point Likert scale. | Competency standards for radiological technologists (RTs) in Korea |
| (McDaniel, Grus et al. 2014) | To synthesize extant literature on primary care (PC) psychology competencies and describe the skills needed to practice in the rapidly changing PC setting, a new practice environment for many psychologists. | Literature review, Conference or workshop, Stakeholder Consultation | Academics, Credentialing and/or certification organisation representatives | - A work group (n=16) participated in a series of presentations via conference call in which the group reviewed existing literature on psychology competencies and PC practice - Group members were then assigned to one of four subgroups in order to compile competencies in that cluster from the literature review - Once the drafts were completed, all but the subgroup leader were assigned to new groups to review and edit the work to allow fresh perspective - The full work group then convened for a two-day meeting in person, during which the group reviewed the full draft document and split into original subgroups to edit competencies based on the large group review - The draft was then submitted for comments by organisations who had representatives on the workgroup. | Competencies for psychology practice in primary care |
| (Misener, Alexander et al. 1997) | To identify competencies needed by nurse leaders in public health programs. | Delphi technique | Practitioners, Employers and/or managers, Credentialing and/or certification organisation representatives | - A convenience sample of members of major public health nursing associations and nurse and non-nurse public health leaders in the USA participated in a five round national Delphi survey to reach consensus on competencies for nurse leaders in public health programs - The round one survey was based on an existing competency framework (Pew Foundation health professions’ competencies) - Four additional rounds produced consensus on 57 competencies organised into four domains. | Competencies for nursing leadership in public health |
| (Moaveni, Gallinaro et al. 2010) | This paper describes the results of a Delphi panel process to gain consensus on a role description and competency framework for family practice registered nurses (FP-RNs) in Ontario. | Interviews, Focus groups, Delphi technique | Practitioners, Academics, Credentialing and/or certification organisation representatives, Practitioners from other professions | - Interviews and focus groups were conducted with FP-RNs and their interprofessional colleagues in order to identify what the exemplary FP-RN does in the family practice setting - These thematic roles were used as a foundation to develop corresponding enabling competency statements, subsequently extracted in a similar manner from recurring content in the narrative data specific to the overarching themes - For phase 2 of the study we convened an inter-professional panel of experts to validate our competency framework by way of modified Delphi technique - Round one and two required participants to evaluate each role description and competency on a five-point Likert scale, with an opportunity to provide feedback and suggest other elements for inclusion - Round three involved discussion in small groups on content of the framework. | A competency framework for family practice registered nurses (FP-RNs) in Ontario |
| (Palermo, Conway et al. 2016) | This study develops competency standards for dietitians in order to substantiate an approach to competency standard development. | Literature review, Focus groups, Delphi technique | Practitioners, Academics, Employers and/or managers, New graduates or students | - Seven focus groups were conducted with 15 employers/practitioners, 5 academics, 8 new graduates (n=28). Text was coded, and codes grouped into categories reflective of the framework. Existing standards and health workforce capability statements were used to transform codes and categories into CS statements. Where an existing standard did not exist, new statements were developed drawing from literature - All authors revised and modified 4 drafts of the CS until they reached consensus - A two-round Delphi study was used to validate the draft competencies Participants were asked to rate each item on a 5-point Likert scale. | Competency standards for dietitians in Australia |
| (Palermo, Capra et al. 2017) | This study aimed to explore the work roles, major tasks and core activities of advanced practice dietitians in Australia to define the Competency Standards for advanced practice. | Focus groups, Interviews | Practitioners, Employers and/or managers | - Four focus groups were conducted with a total of 17 participants (15 dietitian practitioners plus 2 employers) (1 dietitian and 1 non-dietitian) to explore the key purpose, roles and outcomes of practitioners - Data from the focus groups were confirmed with in-depth interviews about their core activities with a purposive sample of individuals recently recognised as Advanced Accredited Practising Dietitians. Data from both focus groups and interviews were analysed to identify key themes - These outcomes were conceptualised within a broad generalist framework to generate revised competency standards. | Advanced practice competency standards for dietetics in Australia |
| (Pastrana, Wenk et al. 2016) | To describe a consensus-based process workshop to develop palliative care competences for medical and nursing schools in Colombia and to present a summary of the findings. | Conference or workshop | Academics, Employers and/or managers, Credentialing and/or certification organisation representatives, Content experts | - A workshop with 36 participants representing 16 medical and 6 nursing schools from 18 universities in Colombia was used to discuss and define PC competencies at the undergraduate level using the International Association for Hospice and Palliative Care (IAHPC) List of Essential Practices (LEP) as guidance. | Palliative care competences for medical and nursing schools in Colombia |
| (Phillips, Ash et al. 2000) | Research involving interviews of new graduate dietitians was conducted to investigate the nature of current dietetic practice, and comparisons were made with the existing competency standards for entry level dietitians. | Interviews | New graduates or students | - Interviews with new graduate dietitians (n=24) were conducted to identify the activities performed by entry-level practitioners, and the underlying attributes requires to perform these activities competently. | Competency standards for entry-level dietitians |
| (Santy, Rogers et al. 2005) | To develop a competency framework for orthopaedic and trauma nursing. | Literature review, Focus groups, Conference or workshop | Practitioners, Academics, Employers and/or managers, Credentialing and/or certification organisation representatives | - The working group ran a series of focus groups to identify common beliefs about orthopaedic and trauma nursing and the care needs of these patients. Thematic analysis was used to develop an interpretation of the nature of practice. Existing literature was incorporated into the interpretation - Workshops were attended by a self-selected group of orthopaedic and trauma nurses working in a variety of settings, led by the same facilitator. The workshops aimed to identify competencies and levels of practice within each of the five core activities. Participants were split into small groups for this. | A competency framework for orthopaedic and trauma nursing |
| (Schofield, Chircop et al. 2018) | To develop consensus on core, national entry-to-practice competencies in public health nursing for undergraduate nursing students and to support these competencies with corresponding online teaching strategies. | Literature review, Delphi technique, Conference or workshop, Survey, World (knowledge) Café | Practitioners, Academics, Employers and/or managers, Credentialing and certification organisation representatives | - A literature scan related to trends and issues in public health nursing practice and education at all levels, existing public health and community nursing competencies and standards and public health elements in current nursing curricula. The task force then engaged in an iterative process of creating, reviewing and revising entry level competencies until a first draft of statements was produced - Thirty-five (n= 35) participants from different areas of PH nursing attended the face to face consultation. Draft competencies were reviewed through a series of knowledge café activities. Small groups of participants discussed and debated the merits of each competency and identified key concepts that were missing or redundant. A large group discussion followed to examine overarching themes identified by the groups. The stakeholder forum input was reviewed by the task force and a second draft of the competencies produced - In the second consultation, an online questionnaire was circulated to deans and directors of the CASN member schools, stakeholder forum participants and colleagues of the task force. Respondents were asked to rate each competency domain and statement as essential, important, somewhat important, not at all important or did not know. | Entry-to-Practice Public Health Nursing Competencies for Undergraduate Nursing Education |
| (Shah, Naidoo et al. 2016) | To develop a comprehensive framework of competency standards for ophthalmic technicians and optometrists, in Mozambique. | Literature review, Delphi technique, Conference or Workshop | Practitioners | - Initial competencies were derived from literature, primary research data and observations from a competency development workshop - A modified Delphi technique was used with a ten-member expert panel consisting of optometrists, ophthalmic technicians and ophthalmologists, all with experience of working in a developing country context was used to further refine the competencies - The first round involved scoring the relevance of two frameworks, one for each cadre, using a nine-point Likert scale with a free-text option to modify any competency or suggest additional competencies. The revised frameworks were subjected to a second round of scoring and free-text comment - The final versions of the agreed frameworks were sent out to the relevant stakeholders. | A competency framework for ophthalmic technicians and optometrists in Mozambique |
| (Suwannaprom, Suttajit et al. 2020) | To explore needs for pharmacy services in the pharmaceutical supply chain and competencies of pharmacists to serve those needs. | Interviews, Stakeholder consultation | Practitioners, Academics, Employers and/or managers, Credentialing and certification organisation representatives | - Semi-structured interviews (n=99) with practitioners and leaders in the field. - Relationships between themes were reorganized and emerged to draw competencies. - Preliminary competency domains and a competency framework for practicing pharmacy services were developed. - The research team members reviewed, discussed and refined the framework. - The competencies and competency framework were presented to professional leaders at a meeting of the Pharmacy Council of Thailand and the Pharmacy Education Consortium of Thailand. Suggestions were harvested from the meeting. - The competency framework was also presented at the Pharmacy Council of Thailand subcommittee on pharmacy workforce meeting and the Thai Pharmacy Education National Conference 2018 for feedback and comments for refining the competency framework. | 1. Proposed pharmacy general competencies within the context of Thailand  2. Proposed pharmacy service specific competencies within the context of Thailand |
| (Tipson and Turner 2002) | To develop an integrated career and competency framework for diabetes nursing. | Values Clarification | Practitioners, Service Users | - More than 50 nurses involved in providing diabetes care in different settings and three service users participated in a workshop involving a values clarification exercise in small groups - Participants were asked to respond to prompts to assess what nurses saw their role/s to be and clarify what competencies are required at different levels of the nursing career. The steering group met to carry out content analysis of the data set in order to identify key themes - Interventions specific to diabetes nursing were then used to clarify specific competencies at each level. | Competency framework for diabetes nursing |
| (Vardanyan, Mosegui et al. 2018) | To evidence the skill-specific, comprehensive core competencies that pharmacists must demonstrate working in humanitarian interventions. | Literature review, Interviews | Practitioners, Employers and/or managers | - A literature search laid the groundwork for the development of interview guides and further analysis of the data - Semi-structured interviews were conducted with expatriate pharmacists and medical coordinators, all of whom have worked in the field of humanitarian assistance (HA). The interviews were recorded, transcribed, and analysed using a content analysis methodology to develop a competency framework for pharmacists in humanitarian assistance. | A competency framework for pharmacists in humanitarian assistance |
| (Williams and Hill 2007) | To review the ACORN competency standards (1999) to ensure their relevancy to current perioperative nursing practice. | Literature review, Delphi technique | Practitioners, Academics | - Purposive and snowball sampling techniques were used to select an expert panel of perioperative nurses across Australia for a Delphi survey. The survey questionnaire was based on the tool used in the original validation project for perioperative nursing competency standards - The first round was an appraisal of the relevancy of the existing competency standards, using a 4-point Likert scale. - The second questionnaire asked participants to appraise the revised competencies. This questionnaire was analysed to identify further themes. The data collected from the second questionnaire indicated a correlation of similar comments and ideas - Correlation of the second-round data was arranged into a third feedback sheet that asked participants to review a second draft of the standards. | ACORN Competency Standards for Perioperative Nurses |
| (Wilson, Callender et al. 2014) | To: a) discuss the benefits of developing interprofessional as well as discipline-specific global health competencies; b) highlight themes that emerged from a preliminary review of existing literature addressing global health competencies and c) review the process used by the CUGH subcommittee to identify two levels of interprofessional global health competencies. | Literature review, Conference or workshop, Survey | Academics | - As a first step in identifying cross-cutting interprofessional competencies, members of the subcommittee reviewed more than 100 published articles and websites pertaining to global health competencies. The domains that were identified in the global competency model developed by the ASPPH were used to guide development of the preliminary template - Following the initial literature review and preparation of the summary tables, subcommittee members proposed interprofessional competencies that should be included in 12 broad competency domains - The subcommittee chair developed a composite list from the competencies identified by members that deleted redundancies and combined similar competency statements - A survey was used to refine the competency standards. In the first stage of competency ratings, subcommittee members indicated whether or not they believed that each of the 83 identified competencies should be included in the final list - In a second competency rating, 11 subcommittee members rated each of the 74 competencies for two levels of students: global citizen and basic operational level. | Interprofessional Global Health Competencies |
| (Wood, Flavell et al. 2009) | To develop an interprofessional competency framework. | Literature review, Conference or workshop, Stakeholder consultation | Academics, Content experts | - The GIFS 2007 competencies and existing frameworks were used to develop a draft IPE framework - The initial draft was presented to IPE and curriculum experts from the BC health authorities and health and human service programs at UBC. Several representatives from these groups participated in an intensive face to face session to review the draft document. The modified document was presented electronically to the group. | British Columbian competency framework for interprofessional collaboration |
| (Yates, Evans et al. 2007) | This project reported in this paper illustrates how a set of competency standard for specialist breast nurses (SBNs) were developed by the National Breast Cancer Centre. | Literature review, Stakeholder consultation, Survey | Practitioners, Academics, Employers and/or managers, Credentialing and/or certification organisation representatives, Service users, Practitioners from other professions | - A focused review of Australian and international literature provided the foundation for the development of a draft set of competencies - A discussion paper based on the review was circulated to 60 key stakeholders by NBCC. Respondents were asked to comment on the relevance and fit of the broad role areas and competency domains for SBN practice and educational requirements for competency SBN practice. A draft set of competencies were developed to reflect key themes - The draft was refined through two further rounds of postal consultation, using a structured response format, where respondents were asked to comment again on the relevance and fit of the competency standard statements, elements and performance criteria - A further, focused literature review was undertaken to identify additional studies providing specific evidence relevant to the domains of practice and competencies identified previously. | Competency Standards for Specialist Breast Nurses in Australia |
| (Zaini, Bin Abdulrahman et al. 2011) | This article describes the development of a competency-based framework for the Kingdom of Saudi Arabia. | Literature review, Stakeholder consultation, Conference or Workshop | Academics, Content experts | - A literature review of national and global competence-based medical frameworks was undertaken - A brain storming session of the project taskforce generated a vision of the competent doctor and formed the basis for a draft competency framework - The draft framework was presented for discussion and comment at the Dean’s committee and sent to an international consultant. The framework was revised based on comments and suggestions. | The Saudi Meds Framework (A competency framework for medical graduates in Saudi Arabia) |
| (Zhang, Meng et al. 2020) | To construct a competency framework specific to specialist critical care nurses in China. | Literature review, Focus groups, Delphi technique, Stakeholder consultation | Practitioners, Practitioners from other professions, Employers and/or managers, Academics | - A literature review and four focus groups with practitioners and practitioners from other professions who worked closely with specialist care critical nurses (n=18) were used to develop the initial competency framework. - Three rounds of a modified Delphi process were conducted with a national panel of experts (n=30), who responded to the survey using a 5-point Likert-type scale to indicate level of agreement with the competency framework. - A 75% threshold for each competency was used to achieve panel consensus. - The final competency framework was reviewed by five external experts. | A competency framework for specialist critical care nurses in China. |
| (Zupanc, Burgess-Limerick et al. 2015) | To supplement existing colonoscopy curriculum documents with a competency framework for colonoscopy training based on cognitive task analysis methods combined with expert review. | Interviews, Observation of practice | Practitioners | - Video recording and a think-aloud protocol were conducted while 20 experienced endoscopists performed colonoscopy procedures - “Cued-recall” interviews were also carried out post-procedure with nine of the endoscopists. Analysis of the resulting transcripts employed the constant comparative coding method within a grounded theory framework - The resulting draft competency framework was modified after review during semi-structured interviews conducted with six expert endoscopists. | A Competency Framework for Colonoscopy Training |

**Supplementary Table 3. Stakeholder engagement in papers which reported conducting a conference or workshop in competency framework development methodology**

| **Lead Author** | **Year** | **Practitioners** | **New Graduates or students** | **Academics** | **Practitioners from other professions** | **Service users** | **Employers and/or managers** | **Policy Influencers** | **Content experts** | **Credentialing and/or certification organisation representatives** |
| --- | --- | --- | --- | --- | --- | --- | --- | --- | --- | --- |
| Albarquoni | 2018 | X |  | X |  |  |  |  |  |  |
| Bobonich & Nolen | 2018 | X |  | X |  |  |  |  |  |  |
| Charles | 2014 |  |  | X |  |  |  |  |  |  |
| Clark | 2011 | X |  |  |  |  |  |  |  |  |
| Davis | 2008 | X |  |  |  | X |  |  |  |  |
| Dunn | 2000 |  |  |  |  |  |  |  |  |  |
| Forsyth | 2018 | X |  | X |  |  |  |  |  | X |
| Halcomb | 2017 |  |  |  |  |  |  |  |  |  |
| Haruta | 2016 | X |  | X |  | X |  |  |  | X |
| Hay | 2007 | X |  | X |  |  |  |  |  |  |
| Homer | 2007 | X | X |  |  |  |  | X |  |  |
| Jacono | 2011 |  | X | X |  |  |  | X |  | X |
| Jenkins & Calzone | 2007 | X |  |  |  |  |  |  |  | X |
| Kiely & Chakman | 1993 | X |  | X |  |  |  |  |  |  |
| Kiely | 2009 | X |  | X |  |  |  |  |  |  |
| Kiely & Slater | 2015 | X |  | X |  |  |  |  |  |  |
| Kiguli | 2014 | X |  | X |  |  |  |  |  |  |
| Leipzig | 2009 | X |  | X |  |  | X |  |  | X |
| McDaniel | 2014 |  |  | X |  |  |  | X |  | X |
| Pastrana | 2016 |  |  | X |  |  |  |  | X | X |
| Santy | 2005 | X |  |  |  |  | X |  |  |  |
| Schofield | 2018 | X |  | X |  |  |  |  |  |  |
| Shah | 2015 |  |  |  |  |  |  |  |  |  |
| Wood | 2009 |  |  | X |  |  |  |  | X |  |
| Yates | 2007 | X |  | X |  |  |  |  |  |  |
| Zaini | 2011 |  |  | X |  |  |  |  |  |  |

**Supplementary Table 4. Stakeholder engagement in papers which reported using the Delphi technique in competency framework development methodology**

| **Lead Author** | **Year** | **Practitioners** | **New Graduates or students** | **Academics** | **Practitioners from other professions** | **Service users** | **Employers and/or managers** | **Policy Makers** | **Content experts** | **Credentialing and/or certification organisation representatives** |
| --- | --- | --- | --- | --- | --- | --- | --- | --- | --- | --- |
| Albarquoni | 2018 | X |  | X |  |  |  |  |  |  |
| Ash | 2015 |  |  | X |  |  | X |  |  |  |
| Attard | 2019b | X |  | X |  | X |  | X | X |  |
| Bobonich & Nolen | 2018 |  |  | X |  |  |  |  |  | X |
| Burns | 2014 | X |  | X |  |  |  |  |  |  |
| Calzone | 2002 | X |  | X |  | X |  | X |  |  |
| Campbell | 2019 | X |  | X |  |  |  |  |  | X |
| Carrico | 2008 | X |  |  |  |  |  |  |  |  |
| Carrougher | 2018 | X |  | X |  |  | X |  |  |  |
| Courtenay | 2018 | X |  | X |  |  | X |  |  |  |
| Esplen | 2020 | X |  | X |  |  |  |  |  |  |
| Galbraith | 2017 | X |  | X |  |  |  |  |  |  |
| Gill | 2017 | X |  | X |  |  | X |  |  |  |
| Greco | 2011 |  |  | X |  |  |  |  |  | X |
| Hay | 2007 | X |  |  | X |  |  |  |  |  |
| Hoxhaj | 2021 | X |  |  |  |  |  |  |  |  |
| Hughes | 2004 | X |  | X |  |  |  |  |  |  |
| Hughes | 2015 | X |  | X |  |  | X |  |  |  |
| Jie & Wanyi | 2018 | X |  | X |  |  | X |  |  |  |
| Kruszewski & Spell | 2018 |  |  | X |  |  |  |  |  |  |
| Liddell | 2017 | X |  | X |  |  |  |  |  |  |
| Misener | 1997 | X |  |  |  |  | X | X |  | X |
| Moaveni | 2010 | X |  | X | X |  |  |  |  | X |
| Palermo | 2016 | X |  | X |  |  |  |  |  |  |
| Schofield | 2018 | X |  | X |  |  |  | X |  | X |
| Shah | 2016 | X |  |  |  |  |  |  |  |  |
| Williams & Hill | 2007 | X |  | X |  |  |  |  |  |  |
| Zhang | 2020 | X |  | X |  |  | X |  |  |  |

**Supplementary Table 5. Stakeholder engagement in papers which reported undertaking stakeholder consultation in competency framework development methodology**

| **Lead Author** | **Year** | **Practitioners** | **New Graduates or students** | **Academics** | **Practitioners from other professions** | **Service users** | **Employers and/or managers** | **Policy Makers** | **Content experts** | **Credentialing and/or certification organisation representatives** |
| --- | --- | --- | --- | --- | --- | --- | --- | --- | --- | --- |
| Albarquoni | 2018 |  |  |  |  |  |  |  | X |  |
| Ash | 2015 | X |  |  |  |  |  |  |  |  |
| Campbell | 2019 |  |  |  |  |  |  |  |  | X |
| Cashin | 2017 | X |  |  |  | X |  | X |  | X |
| Davis | 2008 |  |  |  |  |  |  |  |  | X |
| Dressler | 2006 | X |  | X |  |  |  |  | X | X |
| Halcomb | 2017 |  |  |  |  |  |  |  |  |  |
| Haruta | 2016 | X |  | X |  | X |  |  |  | X |
| Jenkins & Calzone | 2007 | X |  | X |  | X |  |  |  | X |
| Kiely & Chakman | 1993 | X |  | X |  |  |  |  |  | X |
| Kiely | 2009 | X |  | X |  |  |  |  |  | X |
| Kiely | 2000 | X |  | X |  |  |  |  |  | X |
| Kiely & Slater | 2015 | X |  | X |  |  | X |  |  | X |
| Kiguli | 2014 |  | X | X |  | X | X | X |  |  |
| McDaniel | 2014 |  |  | X |  |  |  | X |  | X |
| Santy | 2005 | X |  |  |  |  |  |  |  | X |
| Suwannaprom | 2020 |  |  |  |  |  |  |  |  | X |
| Wood | 2009 |  |  | X |  |  |  |  | X |  |
| Yates | 2007 | X |  | X | X | X | X |  |  |  |
| Zaini | 2011 |  |  | X |  |  |  |  | X |  |
| Zhang | 2020 | X |  |  | X |  | X |  |  |  |

**Supplementary Table 6. Stakeholder engagement in papers which reported conducting interviews in competency framework development methodology**

| **Lead Author** | **Year** | **Practitioners** | **New Graduates or students** | **Academics** | **Practitioners from other professions** | **Service users** | **Employers and/or managers** | **Policy Makers** | **Content experts** | **Credentialing and/or certification organisation representatives** |
| --- | --- | --- | --- | --- | --- | --- | --- | --- | --- | --- |
| Brown | 2012 | X |  |  |  |  | X |  |  |  |
| Canning | 2007 | X |  |  |  |  |  |  |  |  |
| Carter | 2018 | X |  |  |  |  |  |  |  |  |
| Cashin | 2017 | X |  |  |  | X |  |  |  |  |
| Chen | 2013 | X |  | X | X | X |  |  |  |  |
| Dunn | 2000 | X |  |  |  |  |  |  |  |  |
| Esplen | 2020 | X |  |  |  |  |  |  |  |  |
| Ford & McIntyre | 2004 | X |  |  | X |  |  |  |  |  |
| Homer | 2007 | X |  |  |  |  |  |  |  |  |
| Jidkov | 2019 | X |  | X |  |  |  |  | X |  |
| Keijser | 2019 | X | X |  | X | X | X |  |  | X |
| Kiely & Chakman | 1993 |  | X |  |  |  |  |  |  |  |
| Liddell | 2017 | X |  | X |  |  |  |  |  |  |
| Moaveni | 2010 | X |  |  |  |  |  |  |  |  |
| Palermo | 2017 | X |  |  |  |  |  |  |  |  |
| Phillips | 2000 |  | X |  |  |  |  |  |  |  |
| Suwannaprom | 2020 | X |  | X |  |  | X |  |  | X |
| Vardanyan | 2018 | X |  |  |  |  | X |  |  |  |
| Zupanc | 2015 | X |  |  |  |  |  |  |  |  |

**Supplementary Table 7. Stakeholder engagement in papers which reported conducting a survey in competency framework development methodology**

| **Lead Author** | **Year** | **Practitioners** | **New Graduates or students** | **Academics** | **Practitioners from other professions** | **Service users** | **Employers and/or managers** | **Policy Makers** | **Content experts** | **Credentialing and/or certification organisation representatives** |
| --- | --- | --- | --- | --- | --- | --- | --- | --- | --- | --- |
| Al-Haqan | 2020 | X |  |  |  |  |  |  |  |  |
| Brown | 2012 | X |  |  | X |  |  |  |  |  |
| Canning | 2007 | X |  |  |  |  |  |  |  |  |
| Carter | 2018 | X |  |  |  |  |  |  |  |  |
| Cashin | 2017 | X |  |  |  | X |  | X |  | X |
| Curl | 2010 | X |  | X |  |  | X |  |  |  |
| Dressler | 2006 | X |  |  |  |  |  |  |  | X |
| Esplen | 2020 | X |  |  |  |  |  |  |  |  |
| Halcomb | 2017 | X |  |  |  |  |  |  |  |  |
| Homer | 2007 | X |  | X |  |  | X |  |  |  |
| Jacono | 2011 | X |  |  | X |  | X |  | X |  |
| Keijser | 2019 | X | X |  |  | X | X |  |  |  |
| Kiely & Slater | 2015 |  |  |  |  |  | X |  |  |  |
| Lim | 2017 | X |  |  |  |  |  |  |  |  |
| Schofield | 2018 | X |  | X |  |  | X |  |  |  |
| Wilson | 2014 |  |  | X |  |  |  |  |  |  |

**Supplementary Table 8. Stakeholder engagement in papers which reported conducting focus groups in competency framework development methodology**

| **Lead Author** | **Year** | **Practitioners** | **New Graduates or students** | **Academics** | **Practitioners from other professions** | **Service users** | **Employers and/or managers** | **Policy Makers** | **Content experts** | **Credentialing and/or certification organisation representatives** |
| --- | --- | --- | --- | --- | --- | --- | --- | --- | --- | --- |
| Al-Haqan | 2020 | X |  |  |  |  |  |  |  |  |
| Ash | 2015 | X | X |  |  |  |  |  |  |  |
| Attard | 2019b | X |  | X |  | X |  |  | X |  |
| Brown | 2012 | X |  |  |  |  | X |  |  |  |
| Carter | 2018 | X |  |  |  | X |  |  |  |  |
| Esplen | 2020 | X |  |  |  |  |  |  |  |  |
| Ford & McIntyre | 2004 | X |  |  | X |  |  |  |  |  |
| Gill | 2017 | X |  |  |  |  |  |  |  |  |
| Halcomb | 2017 | X |  |  |  |  |  |  |  |  |
| Harding | 2015 | X |  |  |  |  |  |  |  |  |
| Keijser | 2019 | X |  |  |  |  |  |  |  |  |
| Moaveni | 2010 | X |  |  | X |  |  |  |  |  |
| Palermo | 2016 | X | X | X |  |  | X |  |  |  |
| Palermo | 2017 | X |  |  |  |  | X |  |  |  |
| Santy | 2005 | X |  |  |  |  |  |  |  |  |

**Supplementary Table 9. Stakeholder engagement in papers which reported conducting observation of practice in competency framework development methodology**

| **Lead Author** | **Year** | **Practitioners** | **New Graduates or students** | **Academics** | **Practitioners from other professions** | **Service users** | **Employers and/or managers** | **Policy Makers** | **Content experts** | **Credentialing and/or certification organisation representatives** |
| --- | --- | --- | --- | --- | --- | --- | --- | --- | --- | --- |
| Cashin | 2017 | X |  | X |  |  | X | X |  |  |
| Dunn | 2000 | X |  |  |  |  |  |  |  |  |
| Ford & McIntyre | 2004 | X |  |  |  |  |  |  |  |  |
| Homer | 2007 | X |  |  |  |  |  |  |  |  |
| Kiely & Chakman | 1993 | X | X |  |  |  |  |  |  |  |
| Zupanc | 2015 | X |  |  |  |  |  |  |  |  |

**Supplementary Table 10. Stakeholder engagement in papers which reported conducting a values clarification exercise in competency framework development methodology**

| **Lead Author** | **Year** | **Practitioners** | **New Graduates or students** | **Academics** | **Practitioners from other professions** | **Service users** | **Employers and/or managers** | **Policy Makers** | **Content experts** | **Credentialing and/or certification organisation representatives** |
| --- | --- | --- | --- | --- | --- | --- | --- | --- | --- | --- |
| Davis | 2008 | X |  |  |  | X |  |  |  |  |
| Tipson & Turner | 2002 | X |  |  |  | X |  |  |  |  |

**Supplementary Table 11. Stakeholder engagement in papers which reported conducting a World (Knowledge) Café exercise in competency framework development methodology**

| **Lead Author** | **Year** | **Practitioners** | **New Graduates or students** | **Academics** | **Practitioners from other professions** | **Service users** | **Employers and/or managers** | **Policy Makers** | **Content experts** | **Credentialing and/or certification organisation representatives** |
| --- | --- | --- | --- | --- | --- | --- | --- | --- | --- | --- |
| Haruta | 2016 |  |  | X |  |  |  |  |  |  |
| Schofield | 2018 | X |  | X |  |  |  |  |  |  |

**Supplementary Table 12. Stakeholder engagement in papers which reported using the nominal group technique in competency framework development methodology**

| **Lead Author** | **Year** | **Practitioners** | **New Graduates or students** | **Academics** | **Practitioners from other professions** | **Service users** | **Employers and/or managers** | **Policy Makers** | **Content experts** | **Credentialing and/or certification organisation representatives** |
| --- | --- | --- | --- | --- | --- | --- | --- | --- | --- | --- |
| Al-Haqan | 2020 | X |  | X |  |  |  | X |  |  |
| Kirk | 2014 | X |  | X |  | X | X |  |  | X |
